# Monitoring one-carbon metabolism by mass spectrometry to assess liver function and disease

**DOI:** 10.1007/s13105-021-00856-3

**Published:** 2021-12-13

**Authors:** Laura Guerrero, Bruno Sangro, Verónica Ambao, José Ignacio Granero, Antonio Ramos-Fernández, Alberto Paradela, Fernando J. Corrales

**Affiliations:** 1 Functional Proteomics Laboratory, Centro Nacional de Biotecnología-CSIC, Proteored-ISCIII, Darwin 3, 28049 Madrid, Spain; 2 Hepatology Department, University Clinic of Navarra, University of Navarra, 31008 Pamplona, Spain; 3 National Institute for the Study of Liver and Gastrointestinal Diseases (CIBERehd, Carlos III Health Institute), 28029 Madrid, Spain; 4 IdiSNA, Navarra Institute for Health Research, 31008 Pamplona, Spain; 5 Centro de Investigaciones Endocrinológicas “Dr. César Bergadá” (CEDIE) CONICET–FEI–División de Endocrinología, Hospital de Niños R. Gutiérrez, 1330, C1425EFD Buenos Aires, Gallo Argentina; 6 Proteobotics S.L, 28035 Madrid, Spain

**Keywords:** Targeted proteomics, SRM, CPTAC, Biology and Disease Human Proteome Project (B/D-HPP), Liver cancer, Liver injury, One-carbon metabolism

## Abstract

Precision medicine promises to overcome the constraints of the traditional “one-for-all” healthcare approach through a clear understanding of the molecular features of a disease, allowing for innovative and tailored treatments. State-of-the-art proteomics has the potential to accurately explore the human proteome to identify, quantify, and characterize proteins associated with disease progression. There is a pressing need for informative biomarkers to diagnose liver disease early in its course to prevent severe disease for which no efficient treatment is yet available. Here, we propose the concept of a cellular pathway as a functional biomarker, whose monitorization may inform normal and pathological status. We have developed a standardized targeted selected-reaction monitoring assay to detect and quantify 13 enzymes of one-carbon metabolism (1CM). The assay is compliant with Clinical Proteomics Tumor Analysis Consortium (CPTAC) guidelines and has been included in the protein quantification assays that can be accessed through the assay portal at the CPTAC web page. To test the feasibility of the assay, we conducted a retrospective, proof-of-concept study on a collection of liver samples from healthy controls and from patients with cirrhosis or hepatocellular carcinoma (HCC). Our results indicate a significant reconfiguration of 1CM upon HCC development resulting from a process that can already be identified in cirrhosis. Our findings indicate that the systematic and integrated quantification of 1CM enzymes is a promising cell function-based biomarker for patient stratification, although further experiments with larger cohorts are needed to confirm these findings.

**Supplementary Information:**

The online version contains supplementary material available at 10.1007/s13105-021-00856-3.

## Introduction

Hepatocellular carcinoma (HCC) is among the most common malignancies worldwide, imposing a heavy burden on society and health systems [8] and with a steady increase in incidence and mortality [25]. Indeed, liver tumors are the sixth most frequent cancer type and second-leading cancer-associated death [10]. HCC accounts for ~ 90% of liver tumors [29] and is considered as a late complication of chronic liver disease that associates with liver cirrhosis in as much as 80% of cases [9]. The development of HCC is a multistep process, from pre-cancerous, low-grade dysplastic nodules to advanced (stages B and C) HCC (Barcelona Clinic Liver Cancer staging) (reviewed in [14]). Although many genetic and environmental risk factors of HCC are now known [35], clinical outcomes remain dismal as most patients are diagnosed at advanced stages and have poor prognosis (5-year survival rates < 20%) [1]. While preventive strategies such as new-generation antiviral therapies are reducing some of the main risk factors associated with HCC (e.g., HBV and HCV infections), other etiologies are emerging such as non-alcoholic steatohepatitis, which is often linked to obesity and diabetes [36]. The lack of robust methods for the early detection and treatment of HCC that can be tolerated by patients with advanced chronic liver disease raises an urgent need to investigate the molecular basis of the disease [23].

The progression of HCC is complex and is based on two main factors: (1) Progression of chronic tissular damage induced by viral infections, metabolic alterations or toxins [3, 29]. This process involves liver inflammation and regeneration that must be finely tuned, as their deregulation leads to fibrosis, cirrhosis and, ultimately, HCC. (2) Genetic alterations involving oncogenes and/or tumor suppressors that lead to the impairment of central cellular pathways such as Wnt, β-catenin, VEGFR/EGFR, PI3K/Akt/mTOR, JAK/STAT, or MAPK [14]. This knowledge has spurred researchers to explore potential HCC oncogenic drivers [28], but unfortunately, this scientific progress has not yet translated into better management of liver cancer [15]. Several promising HCC biomarkers are under investigation [5], but the molecular hallmarks and mediators of HCC progression remain to be identified to fulfill some of the unmet needs, including a better understanding of tumor heterogeneity, integration of molecular subtypes into clinical staging, and clinical indicators to predict treatment response and for early detection/surveillance.

**Fig. 1:**
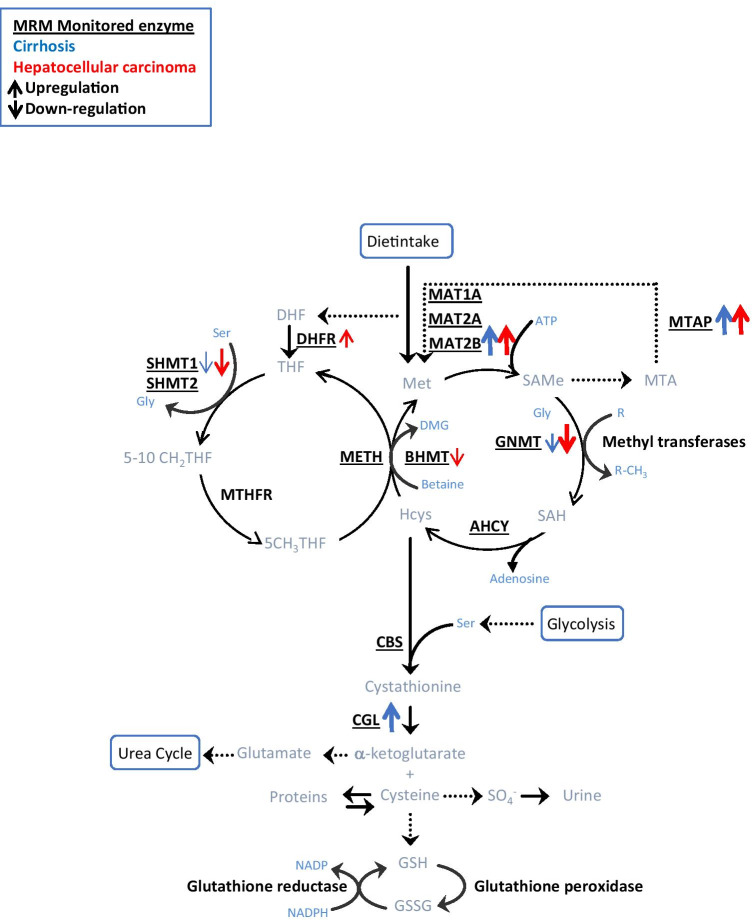
Schematic representation of one-carbon metabolism. The enzymes quantified in this study are highlighted. The arrows represent the significant changes observed (*q* < 0.05) in cirrhosis (blue) and hepatocellualr carconoma (HCC) (red)

Metabolic remodeling is a common feature of most liver ailments, from steatosis to HCC, in which transformed hepatocytes re-shape their metabolism according to their specific proliferative requirements––a condition first described by Warburg [32]. One-carbon metabolism (1CM) is widely recognized as a key metabolic regulatory node to preserve the quiescent and differentiated state of hepatocytes [19] (Fig. 1). Based on its principal role in the regulation of the methylation capacity of the cell, 1CM is considered as a link between intermediate metabolism and epigenetic regulation [20], and its dysregulation is a common finding in many HCC targeted and proteome-wide analyses [11, 26]. In addition to the growing evidence associating the impairment in methionine adenosyltransferase (MAT) enzymes with liver carcinogenesis [17, 26], other 1CM enzymes, including methylthioadenosine phosphorylase (MTAP) might also participate in the progression of HCC [2]. Given the relevance of 1CM to the maintenance of hepatocyte homeostasis, the systematic measurement of participating enzymes has been proposed as a good multi-parameter test for liver function and differentiation assessment.

The identification of biological landmarks to assess the health status of individuals forms the basis of modern precision medicine efforts, providing molecular evidence of the onset and progression of a disease or pharmacological susceptibility, thus allowing for tailored healthcare approaches. Against this background, proteomics is providing new insights into proteins associated with disease that can be systematically measured to guide patient stratification [31]. Multiple-reaction monitoring (MRM)-based strategies are an attractive alternative to more established methods commonly based on the use of affinity reagents (i.e., western blotting or ELISA), and offer a fast and easy methodology that is not limited by the availability of specific antibodies. Additionally, its multiplexing capacity allows for monitoring multiple target proteins in a single liquid chromatography coupled to mass spectrometry (LC–MS) run in a large number of samples, making it the method of choice in biomarker verification and validation pipelines [7]. Indeed, liver research has greatly benefited from large-scale proteome studies, which have aided in the discovery of proteins relevant for the development of innovative clinical strategies [20]. Along this line, our group recently introduced the concept of functional biomarkers, defined here as a panel of functionally related proteins that, for instance, integrate into a metabolic or signaling pathway, adding a second level of information to complement the up- or downregulation of individual proteins. Based on this concept, we have shown that the systematic measurement of 1CM enzymes using selected-reaction monitoring (SRM)/MRM targeted proteomics provides precise information about the specific configuration of this metabolic pathway in liver diseases and HCC in mice [21].

In the present study, as part of the liver team of the Biology and Disease Human Proteome Project (https://www.hupo.org/human-proteome-project), we developed a standardized SRM assay to detect and quantify 13 1CM enzymes in human liver. The assays are compliant with Clinical Proteomics Tumor Analysis Consortium (CPTAC) guidelines and can be accessed through the assay portal at the CPTAC web page. We also conducted a retrospective, proof-of-concept study on a collection of human liver samples from healthy controls and from patients with cirrhosis or HCC to test the clinical use of the assay. Our results indicate a significant reconfiguration of 1CM upon HCC development resulting from a process that can already be identified in cirrhosis. These findings suggest that the systematic and integrated quantification of 1CM enzymes is a valuable resource for the prognosis and follow-up of HCC and of patients with chronic liver disease at risk of HCC.

## Materials and methods

### Biological specimens

Liver samples were collected from patients with liver disease and from asymptomatic (control) patients. Control liver samples (*n* = 28) were obtained from patients with a hydatid cyst, with no additional manifestations of functional or morphological alterations in the liver. Cirrhotic liver samples (*n* = 15) and liver tumors (*n* = 19) were obtained at the time of liver transplantation or tumor resection, respectively. Upon extraction, samples were flash frozen in liquid nitrogen and stored at − 80 °C until use. The present study was approved by the human research review committee of the University Hospital of Navarra, and informed consent was obtained from all patients enrolled in the study. The study was conducted in compliance with the ethical standards formulated in the Helsinki Declaration of 1996 (revised in 2000).

### Sample preparation

Liver specimens were thawed and disrupted mechanically using a Potter–Elvehjem homogenizer in 7 M urea, 2 M thiourea, 4% CHAPS (3-[(3-cholamidopropyl) dimethylammonio]-1-propanesulfonate), 40 mM dithiothreitol (DTT) at pH 7.7. After centrifugation at 10,000 × *g* for 5 min, the protein concentration of the saved supernatant was measured using the Pierce 660-nm Protein Assay (ThermoFisher Scientific). Proteins were precipitated with methanol/chloroform/water (4:1:3 vol.), and the resulting pellet was precipitated again with methanol (4 vol.). Samples were centrifuged, and the precipitated proteins were evaporated to dryness in a Speed Vac concentrator. Proteins were resuspended and reduced in 8 M urea, 100 mM ammonium bicarbonate, 10 mM DTT, pH 8, for 1 h at 37 °C. Free reduced cysteine thiols were alkylated with 50 mM iodoacetamide for 45 min at room temperature in the dark. Subsequently, samples were diluted fourfold with water to reduce the urea concentration to 2 M. Trypsin was then added to a final ratio of 1:25 (trypsin/protein), and the proteolytic reaction was incubated overnight at 37 °C. Tryptic peptides were evaporated in a Speed Vac concentrator, resuspended in 1% trifluoroacetic acid (TFA) and desalted on C18 Stage Tips (ZipTip, Merck-Millipore). Peptide concentration was determined using a Qubit 2.0 fluorometer (ThermoFisher Scientific).

### Definition of the MRM method

To study 1CM in human liver, the abundance of 13 participating enzymes was initially selected: GNMT, AHCY, CBS, CGL, DHFR, MAT1A, MAT2A, MAT2B, MTAP, BHMT, SHMT1, SHMT2, and METH. The selection of proteotypic peptides for inclusion in the MRM development combined in-house shotgun proteomics-based experimental data with public information from the Human Proteome Project at SRMAtlas (http://www.srmatlas.org) [13] and PeptideAtlas (http://www.peptideatlas.org) [6] (Supplementary Table 1). In-house data were generated combining shotgun analysis of human liver samples and of recombinant versions of 10 of the 13 1CM enzymes (recombinant SHMT1 and 2 and METH were not available). In the second case, tryptic digests were spiked into a complex matrix (trypsin-digested *Xanthophyllomyces dendrorhous* proteome) before LC–MS/MS analysis. Detected peptides were then combined with the SRMAtlas and PeptideAtlas candidates, and 45 peptides were selected according to the following criteria: (1) maximum of 4 peptides/protein; (2) common candidates from experimental data and databases; (3) detection Mascot score above 28 for experimental candidates; (4) candidates from databases only, if no peptide was detected by MS; (5) peptide length 8–25 residues; (6) no missed cleavages; and (7) peptides with Met, Trp, or other amino acids that might be modified either in the cellular environment or during the analysis were avoided if alternative peptides were available. To monitor the selected peptide panel, parameters were set on a preliminary MRM analysis, resulting in 40 of 45 positive detections. It is worth noting the complementarity of the different sources of information used: while 21 peptides were common, 14 and 5 peptides were contributed solely by experimental and public data, respectively, to the final method (Supplementary Fig. 1), which increases the likelihood of detection of the selected proteins. Despite the high degree of correlation found between our experimental data and data from the SRMAtlas and PeptideAtlas, we observed several inconsistencies when comparing the transition intensities for a given peptide, likely due to the use of different mass spectrometers used.

To generate the experimental peptide library, 20 pmol of each recombinant protein was incubated in 8 M urea, 100 mM ammonium bicarbonate, 10 mM DTT, pH 8, alkylated with 50 mM iodoacetamide for 45 min, diluted with water to 2 M urea, and digested with trypsin (1/25 ratio trypsin/protein) at 37 °C for 12 h. Tryptic peptides were dried in a Speed Vac, resuspended in 0.1% formic acid in water and desalted on C18 Stage Tips. Subsequently, 100 fmol of the peptides was mixed with 1 μg of a complex peptide background (a digested proteome obtained from *Xantophyllomyces dendrorhous*) for analysis using a quadrupole-time-of-flight mass spectrometer (SCIEX Triple-TOF 5600) coupled to a liquid nanochromatography system (Eksigent Technologies nanoLC Ultra 1D plus). Peptides were resolved on a reversed phase C18 column (1.7- μm particle size, 130 Å pore size, 75 μm I.D. × 15 cm) in a 250-min gradient at a flow rate of 250 nL/min. Gradient conditions were as follows: from 5 to 30% of acetonitrile (ACN) in 180 min, 60% of ACN in 20 min, then 95% of ACN was achieved in 15 min and maintained during 10 min before restoring initial conditions (5% ACN). MS1 and MS2 spectra were acquired in data-dependent acquisition mode. MS1 scan acquisition time was 250 ms, and 25 precursors per spectrum were automatically selected according to the signal intensity, isolated and fragmented for 100 ms/precursor. Total cycle time was 2.8 s. Raw data were processed, and mgf files were generated with PeakView v1.1. MS1 and MS2 spectral data were used to launch a database search using Mascot v2.5.0 (MatrixScience) as the search engine against the UniprotKB human reference proteome database (UP000000589 reviewed, 2019, with 20,239 entries), concatenated to the corresponding decoy version. Static modification was C carbamidomethylation (+ 57.021464) and for stable isotope-labeled (SIL) peptides K + 8.014199 and R + 10.008269. Results were filtered with a false discovery rate (FDR) < 1% at peptide and protein level.

### Peptide synthesis and purification

Light and heavy versions of the selected peptides were synthetized using standard F-moc chemistry. We used ^13^C and ^15^ N lysine and arginine for SIL heavy peptides, resulting in an 8- and 10-Da mass shift, respectively, compared with their light counterparts. Cys residues were blocked with 50 mM iodoacetamide for 1 h at 37 °C. Synthesized peptides were purified on a C18 reversed phase column with a 0–65% ACN gradient at 2 ml/min (JASCO Pu-2089 Plus pump coupled to a JASCO UV-2077 detector). The UV detector was set at 214 and 280 nm to monitor eluting peptides. Main chromatographic peaks were collected and re-analyzed for purity assessment by high resolution analytical LC on a Scharlau C18 column (5- μm particle size, 2 mm I.D. × 25 cm) using a 40-min 0–70% ACN gradient at 250 μl/min at 40 °C (Ultimate 3000 HPLC). The minimal purity of all peptide preparations was set as > 90% pure. Molecular weight and peptide purity were further assessed by MALDI TOF/TOF analysis (SCIEX 4800). Peptide quantification was done by amino acid analysis at the Protein Chemistry core facility of the Biological Research Center (CIB-CSIC; Madrid, Spain).

### nLC-MS/MS analysis

MRM analyses were performed with 1 μg total peptide amount as determined on a Qubit 2.0 fluorimeter. Peptides were loaded into a C18 PepMap trapping column (5-μm particle size, 100 μm I.D. × 5 cm; ThermoFisher Scientific) at 2 μL/min flow rate of 0.1% FA and then separated on a C18 column (3-μm particle size 120 Å pore size, 75 μm I.D. × 15.2 cm) (Nanoseparations, Nieuwkoop, The Netherlands). Elution was achieved with a 60-min stepwise gradient of ACN in 0.1% FA: from 2 to 40% ACN in 42 min, 40 to 95% ACN in 7 min, and 3 min in 95% ACN before re-equilibration in 2% ACN. Peptide separation was performed at 300 nL/min and 40 °C. MS/MS analyses were done on a 5500 QTRAP triple-quadrupole mass spectrometer setting a dwell time of 20 ms (for non-scheduled methods) and a declustering potential of 80 V. Optimization of additional settings as well as the standardization of the method are described in detail in the Results section.

### Data processing

Raw SRM data files were analyzed with Skyline software (v20.1.0.31), and the peak selection in the chromatograms for each peptide was manually curated [22]. Transitions showing some interference in peak area were excluded. The intensity area of each peak was automatically calculated by the software considering the value as the ratio unlabeled/SIL precursors.

### Statistics

Raw peak area measurements were log2-transformed, normalized using linear models accounting for heavy peptide area measurements, and aggregated at each sample into protein abundance estimators using weighted sums. Linear modeling on normalized protein log abundance values was used to assess the significance of clinical condition effects while correcting for subject effects. *p* values were computed using *F* tests, and FDR control was used for multiple testing corrections. Protein abundance estimators were centered and scaled before cluster analysis and principal component analysis (PCA). Hierarchical clustering of protein abundance profiles was conducted using Ward’s method on a Euclidean distance matrix. The number of protein clusters was selected by manual inspection of the resulting dendrogram. Four machine learning algorithms for which a multiclass supervised classification implementation is available were selected: linear discriminant analysis (LDA), quadratic discriminant analysis (QDA), multinomial logistic regression, and random forests. Given the limitations imposed by size of the data set, all algorithms were run with sensible defaults and no metaparameter tuning, and model complexity control was preferred over regularization. Proteins were ranked by ANOVA *p* value, and panels of increasing complexity containing data from 1 to 13 of the 1CM proteins were used to build models. Out-of-sample error estimates for each panel size and algorithm were obtained by leave-one-out cross-validation.

## Results and discussion

### Standardization of the MRM quantification method

Once experimental parameters for peptide isolation and analysis were fixed, we sought to optimize and standardize the method to ensure assay accuracy, reliability, and reproducibility. To this end, we followed the Clinical Proteomics Tumor Analysis Consortium (CPTAC) guidelines [4]. First, several collision energy (CE) values were tested to improve the sensitivity by optimizing the transition intensities of each peptide. This process significantly increased the required number of MS/MS scans per precursor and, therefore, the selected proteins were assayed individually to prevent long duty cycles. The analysis was performed using 250 fmol of each synthetic peptide spiked into a 1-μg *Escherichia coli* extract digested with trypsin, resulting in optimal CE values (Supplementary Table 2). Furthermore, a scheduled method (± 2.5 min window) was set with Skyline using data from the analysis of 100 fmol of light and SIL synthetic peptides spiked into a 1-μg *E. coli* digest as background. Scanning each precursor in a narrow (300 s) window around the expected elution time optimized the shape of the chromatographic peak and its quantification by increasing the signal/noise ratio. To evaluate the specificity of the method and to rule out the effect of electronic or biological interference from the matrix, we used a tryptic digest of Huh7 cells as background––a complex matrix that resembles the proteome of liver samples that will be used during the actual experiments, but in which the expression of the monitored proteins is negligible [21]. A Huh7 extract (1 μg) containing 100 fmol of the mixture of light and SIL synthetic peptides was analyzed. As expected, no detection or very weak signals for all precursors was observed in the non-spiked Huh7 sample, whereas signals for the co-eluting pairs of light and light-SIL peptides were unequivocally detected in the equivalent spiked samples (Supplementary Fig. 2). The results of the interference assay indicated that the observed signals can be reliably assigned to the expected precursors.

**Fig. 2:**
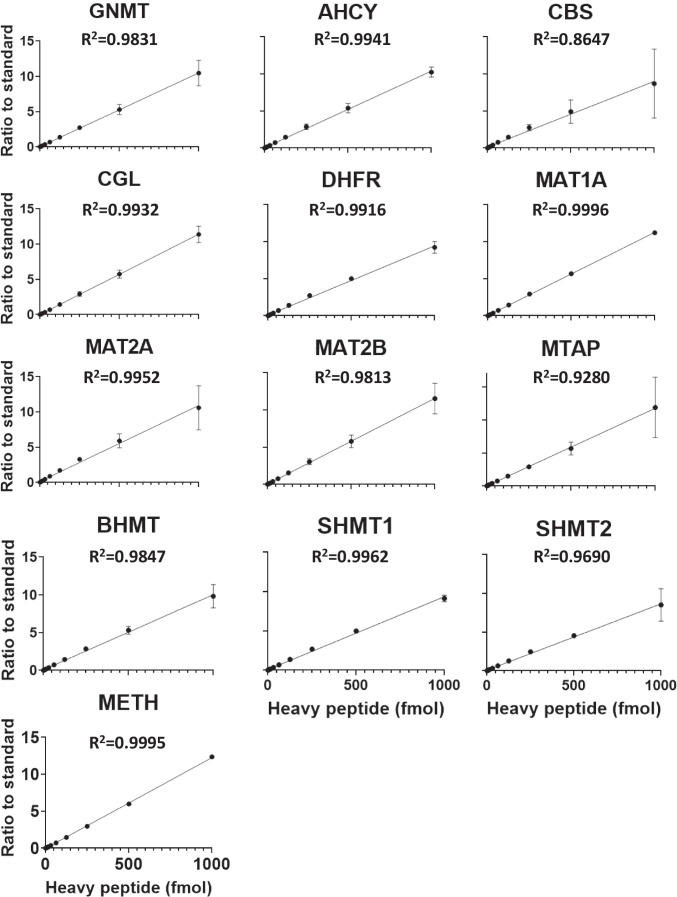
Linearity of one-carbon metabolism (enzymes quantification by multiple-reaction monitoring. An estimation of the linear range of one-carbon metabolism enzyme quantification was determined by averaging the ratio values of the peptides monitored for each protein

The next goal was to define the linear range, the lower limit of detection (LLOD), and the lower limit of quantification (LLOQ) of the assay. Serial dilutions of the SIL peptide panel spanning more than three-orders of magnitude (0.5–1000 fmol) were spiked into the Huh7 digest together with a fixed concentration of the unlabeled synthetic peptides (100 fmol); the area ratio SIL/light peptide was then calculated. The LLOD was determined from three independent blank matrix sample runs as the average plus three times the standard deviation of the blank signal (*n* = 3). When no signal was detected in the blanks, the LLOD was estimated using the standard deviation of the signal detected in the lowest spiked sample. The LLOQ was defined as the lowest concentration of peptide at which the coefficient of variation (CV) of the measured signal was < 20%. For most precursors the LLOD ranged between 0.5 and 4 fmol and LLOQ between 0.5 and 1 fmol (Supplementary Table 3). In some cases, the LLOQ, although very close to, was lower than the LLOD, likely resulting from the different arithmetic measures used for their calculation. The LLOD and LLOQ of a given protein will be that of the corresponding monitored peptide with higher LLOD and LLOQ values. Linearity across concentrations spanning four-orders of magnitude was assessed for the 40 monitored peptides (*R*^2^ < 0.9) (Supplementary Fig. 3). Quantification linearity at the protein level was determined by averaging the ratio values of the peptides monitored for each protein. The linear fitting of the experimental results was done according to CPTAC recommendations and yielded *R*^2^ values > 0.96 in almost all cases (with the exception of 2 peptides, as indicated in Supplementary Table 3), indicating the suitability of the quantification method across the assayed concentration range (Fig. 2).

To estimate the total variability of the LC–MS/MS experiment, intra- and inter-assay variabilities were measured across five consecutive days. The sample used was a Huh7 digest spiked with three different concentrations of each heavy synthetic peptide at low (5 fmol), medium (50 fmol), and high (500 fmol) concentration. Light synthetic peptides were added at a fixed amount (30 fmol). The analysis was done in triplicate. Intra-assay variability was defined as the average of the CV of the three replicates on each of the 5 days. Inter-assay variability was calculated at each concentration as the average of the CV of the first injection across the 5 days, the CV of the second injection across the 5 days, and the CV of the third injection across the 5 days. The total CV was calculated as follows:

$$\mathrm{CV}t=\sqrt{{\left(\mathrm{average intra}-\mathrm{assay} \mathrm{CV}\right)}^{2}+{\left(\mathrm{average inter}-\mathrm{assay CV}\right)}^{2}}$$

**Fig. 3:**
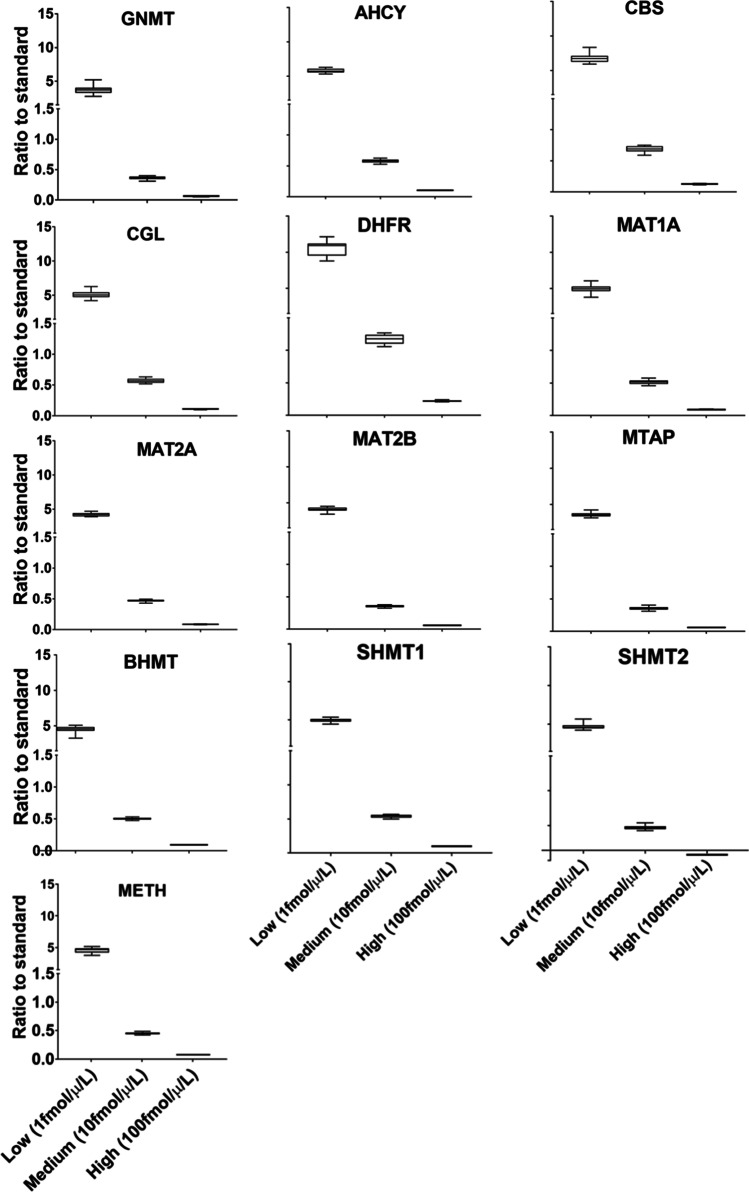
Reproducibility of the quantitation assay for one-carbon metabolism enzymes. Variability of the LC–MS/MS experiment was assessed by measuring the intra- and inter-assay variability across five consecutive days. Intra-assay variability was defined as the averages of the coefficient of variation (CV) of the three replicates on each of the 5 days. The inter-assay variability was calculated at each concentration as the average of the CV of the first injection across the 5 days, the CV of the second injection across the 5 days, and the CV of the third injection across the 5 days. Peptides were accepted when the total CV was < 20%. To estimate the reproducibility of the quantification at the protein level, the total CVs of the corresponding peptides at each concentration were averaged and were always lower than 20%

Peptides were accepted when the intra-, inter-assay, and total CVs were below 20%. For the 23 peptides that met these criteria, the CV value ranges were 0.23–12.86, 1.04–18.27, and 1.77–18.35 for intra-, inter-assay, and total CV, respectively (Supplementary Fig. 4), with larger CV values at lower concentrations. To estimate the reproducibility of the quantification at the protein level, the total CVs of the corresponding peptides at each concentration were averaged (Fig. 3). CVs were consistently below 20%, and the ratio values were the expected ones at each concentration except for DHFR, which was always higher than expected likely because of deviations in the quantification of the standard peptides. In summary, the transitions included in the final method were those allowing protein linear quantifications across more than three-orders of magnitude (0.5–1000 fmol), with LLOD = 0.5–4 fmol, LLOQ = 0.5–1 fmol, and CV values < 20% (Table 1).

**Fig. 4:**
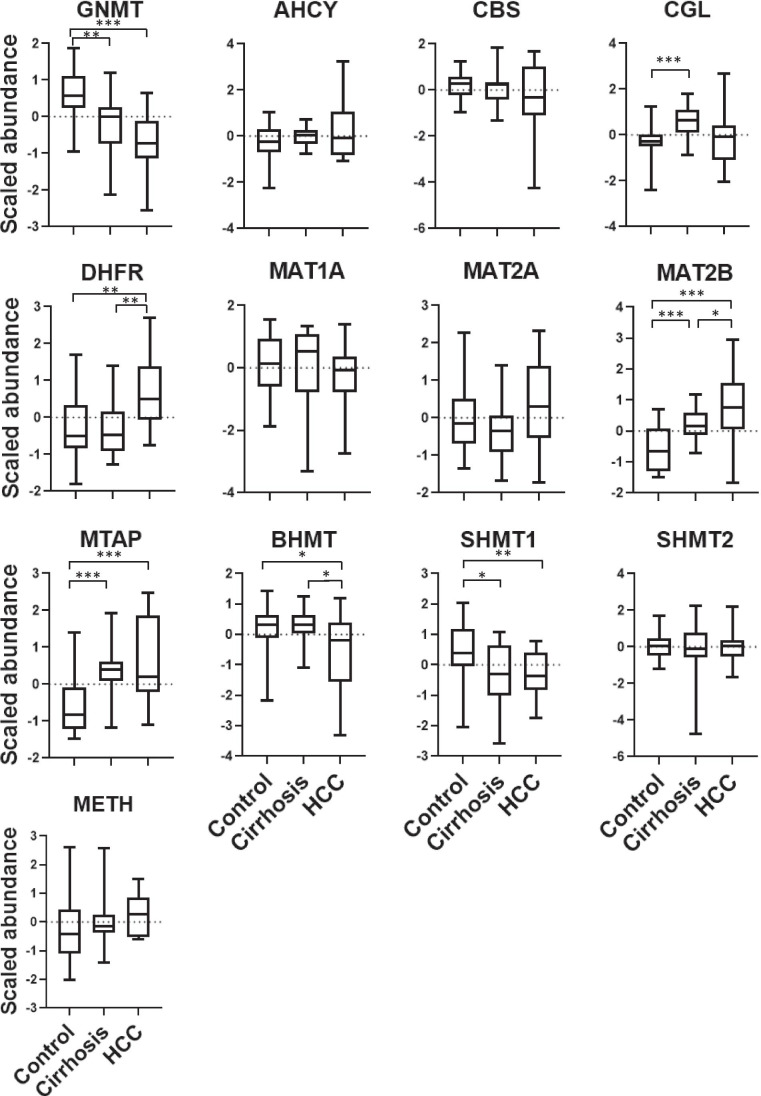
Quantification of one-carbon metabolism enzymes in human liver diseases. The multiple-reaction monitoring method allowing quantitation of 13 one-carbon metabolism enzymes was tested on a panel of human liver samples including healthy (control), cirrhosis, and HCC cases. All proteins were detected across the analyzed samples, and significant differences were observed in some cases (indicated by *p < 0.05, **p < 0.01, ***p < 0.001)

**Fig. 5:**
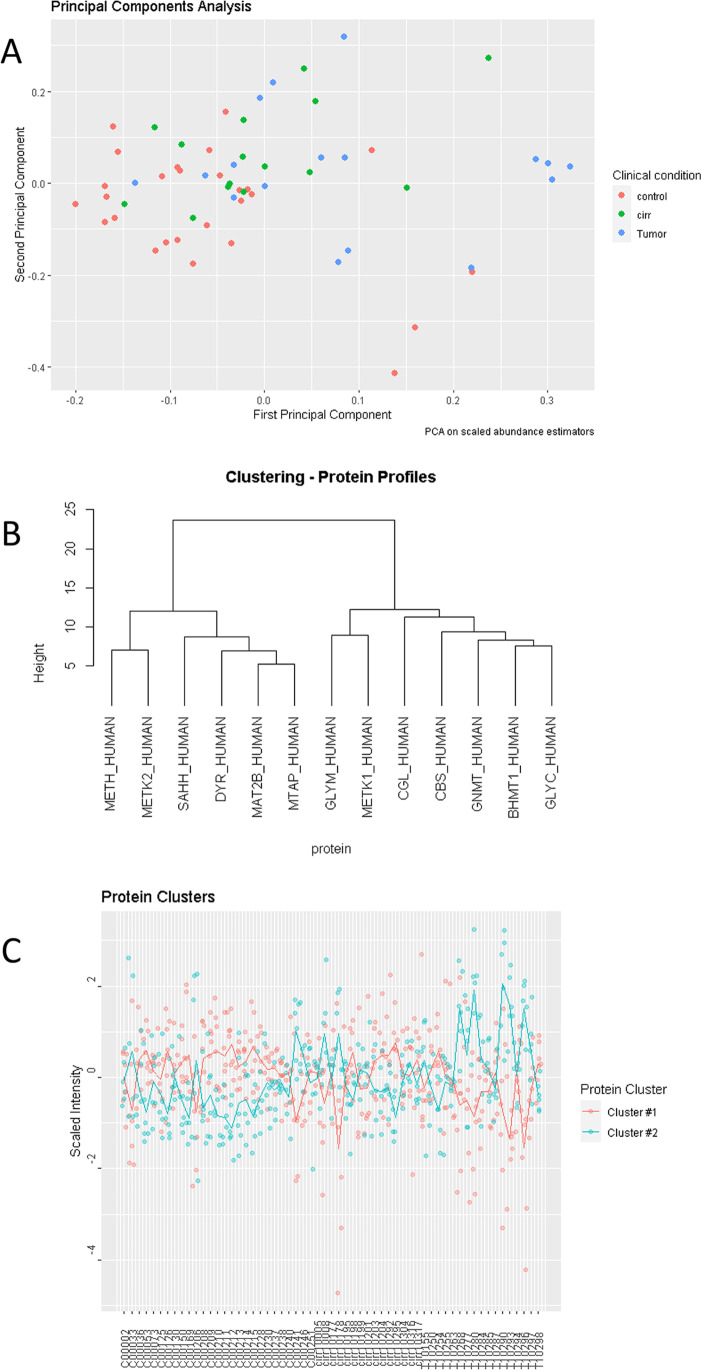
Analysis of one-carbon metabolism allows for liver disease stratification. Principal component analysis of the grouping of control and hepatocellular carcinoma (HCC) samples and the intermediate position of cirrhosis samples (**A**). The monitored proteins grouped into two well-differentiated clusters (**B**) according to their abundance in the set of samples analyzed here. Cluster 1 tended to show higher expression in controls whereas cluster 2 was over-represented in HCC and intermediate profiles were found in cirrhosis (**C**)

To test the performance of the MRM method using real-world samples, we conducted a pilot study of human liver samples from control (healthy) individuals and from patients with cirrhosis or HCC. We also aimed to assess the applicability of the method by defining the specific 1CM pathway profile in liver disorders based on the abundance changes of the participating enzymes and, ultimately, to demonstrate the proof-of-principle of the stratification capacity of 1CM as a functional biomarker. All proteins were detected in liver extracts, and the relative abundances were in good agreement with those published in PaxDB [33] and the Human ProteinAtlas [30] resources. Protein detection was directly correlated with the reported values of cellular abundance, suggesting low levels for DHFR and METH and even less for MTAP, which was close to the LLOD, likely explaining their non-detection in some samples (Supplementary Fig. 5). Significant differences were observed for GNMT, MAT2B, MTAP, DHFR, CGL, SHMT1, and BHMT; specifically, changes (*q* < 0.05) in GNMT, MAT2B, MTAP, DHFR, and SHMT1 were observed in HCC versus control; MTAP, CGL, MAT2B, and GNMT in cirrhosis versus control; and DHFR in HCC versus cirrhosis (Fig. 4). Of note, for most proteins even if no statistical significance was achieved, the expected differential trend was observed across normal, cirrhosis, and tumor groups. Supporting these observations, PCA analysis revealed that HCC and controls could be differentiated by monitoring 1CM enzymes, whereas cirrhosis appeared to represent an intermediate condition, as might be expected from a pretumoral stage (Fig. 5A). The monitored proteins grouped into two clusters with well-differentiated behavior across samples: cluster 1 (GNMT, CBS, CGL, MAT1, BHMT1, SHMT1, and SHMT2) was over-represented in normal liver, and cluster 2 (AHCY, DHFR, MAT2A, MAT2B, MTAP, and METH) was upregulated in HCC (Fig. 5B, C). Of note, cluster 1 comprised proteins with enhanced expression in the liver whereas cluster 2 integrated enzymes with ubiquitous tissue expression according to Protein Atlas. This distribution suggested 1CM reprogramming from a normal liver-specific phenotype to a survival configuration similar to that found in other tissues, which may indicate the loss of hepatocyte differentiation. For instance, it is known that MAT1A is almost exclusively expressed in adult liver, whereas MAT2A and the regulatory MAT2B subunit catalyze the synthesis of adenosylmethionine (AdoMet) in fetal liver and non-hepatic tissues. However, MAT1A is downregulated and progressively replaced by MAT2A in advanced cirrhosis and HCC. AdoMet homeostasis must be finely tuned as any imbalance in its metabolic flux leads to liver proliferation and cancer [16]. In addition to the regulation of the methyl group balance, folate metabolism is also important in cancer cell biology. It is well recognized that tetrahydrofolate has a central role as a coenzyme in different transmethylation reactions (including the recycling of homocysteine to methionine catalyzed by METH), as well as in purine and pyrimidine nucleotide synthesis pathways, which are essential in the synthesis, repair, and replication of DNA. Accordingly, folate metabolism is needed to maintain normal cell growth, especially in highly proliferative cells such as tumoral cells. It is worth noting that one of the most widely used anticancer drugs is methotrexate, which is polyglutamylated inside the cell and becomes a competitive inhibitor of DHFR and other enzymes involved in purine and pyrimidine nucleotide synthesis. Changes in the levels of DHFR and SHMT1 reported here suggest different susceptibilities to MTX therapy, and might provide an index to follow-up patient response after treatment [12].

### Quantification of 1CM enzymes in liver samples from cirrhotic and HCC cases

**Fig. 6:**
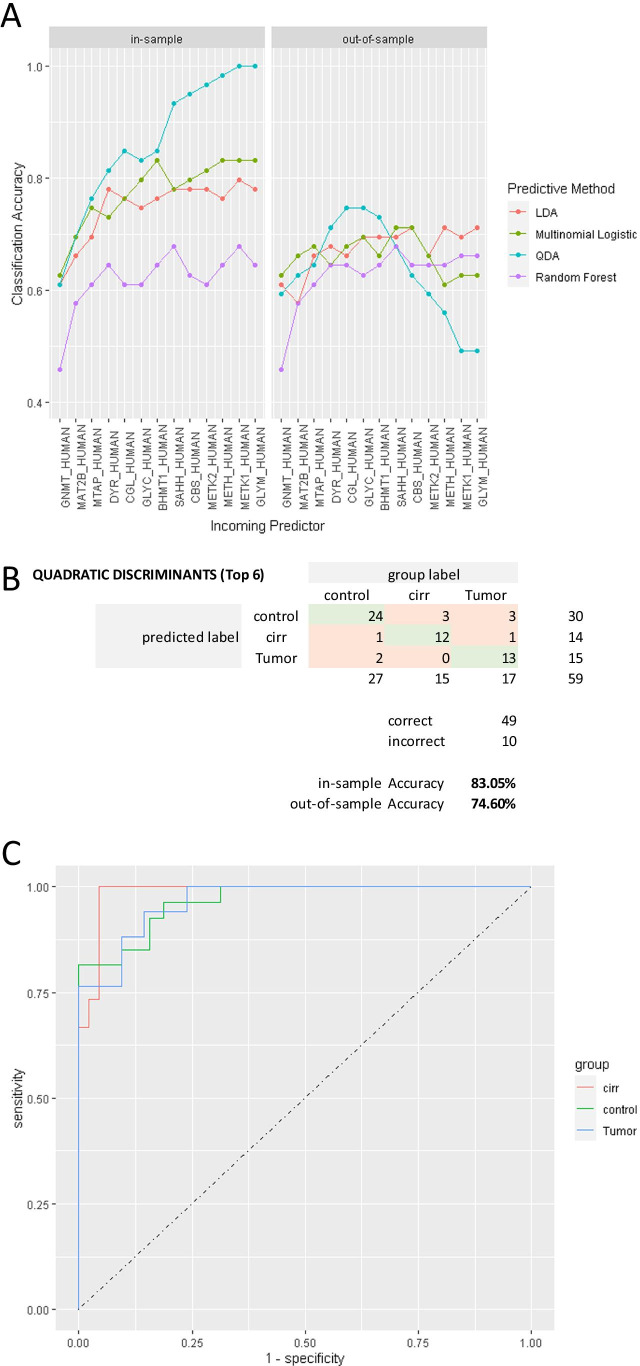
Machine learning-based predictive modeling. Different prediction methods were tested: linear discriminant analysis (LDA), multinomial logistics, quadratic discriminant analysis (QDA), and random forest. The optimal discrimination across the different clinical conditions included in the study was achieved with a panel of six one-carbon metabolism enzymes (GNMT, MAT2B, MTAP, DYR, CGL, GLYC) (**A**). In-sample and out-of-sample accuracies were 83.05% and 74.60% respectively (**B**). Receiver operating characteristic curves showed very good sensitivity/specificity tradeoff with area under the curve values of 0.9664, 0.9864, and 0.9664 for control, cirrhosis (cirr), and hepatocellular carcinoma (tumor), respectively (**C**)

Given the important role of 1CM in liver differentiation and the capacity of our method to measure its impairment, we next questioned whether the quantification of 1CM enzymes may have diagnostic and/or prognostic capacity in monitoring liver function and disease. Supporting this idea, GNMT, CGL, BHMT, and SHMT1 have been considered as favorable prognostic factors for HCC in the Human Protein Atlas, based on transcriptomic and proteomic profiling of control and diseases tissue samples and of cell lines. By contrast, MAT2A and MTAP are considered unfavorable prognostic factors for HCC, in good agreement with our findings. We used a predictive modeling approach to more carefully assess the potential of this metabolic pathway for discerning clinical conditions using protein abundance measurements. Panels of increasing numbers of protein biomarkers with decreasing significance were used to build models using four different prediction methods (see Methods). Limiting the panel size was important to avoid overfitting. The best performance (Fig. 6A) was obtained using a QDA model with a panel comprising six proteins (GNMT, MAT2B, MTAP, DYR, CGL, GLYC). A confusion table for the selected model is shown in Fig. 6B. Estimates of classification accuracy were 83.5% (in-sample) and 74.6% (out-of-sample). Interestingly, the classification accuracy was fairly homogeneous across all clinical conditions, suggesting that no clinical condition is particularly likely to be confounded with another specific condition. Receiver operating characteristic curve analysis showed very good sensitivity/specificity tradeoff, with area under the curve values of 0.9664, 0.9864, and 0.9664 for control, cirrhosis, and HCC respectively (Fig. 6C).

## Conclusions

We have developed and standardized an MRM method to quantify 1CM enzymes in human samples and tested it in a proof-of-concept experiment that illustrates the reconfiguration of 1CM in human cirrhosis and HCC. Our data suggest that methionine is mainly used in transformed hepatocytes for protein synthesis as a requirement for cell growth and proliferation [34]. This hypothesis is supported by studies showing that modulation of protein synthesis is central for HCC response to treatment with the multikinase inhibitor sorafenib [27]. Moreover, the upregulation of methionyl-tRNA-synthetase is considered as an unfavorable prognostic factor for HCC in the Human Protein Atlas. By contrast, the synthetic capacity of AdoMet is decreased, ensuring the maintenance of a basal flux to allow cell survival and preventing excessive ATP expense (the three phosphate groups of an ATP are needed to synthesize a single AdoMet molecule). Regulation of ATP utilization in hepatocytes would prevent nicotinamide adenine dinucleotide depletion and mitochondrial de-energization [24]. Thus, the participating enzymes of 1CM can be considered as a cell function-based biomarker with potential for liver disease stratification, although further experiments including larger cohorts are needed to confirm our preliminary findings.

**Table 1:**
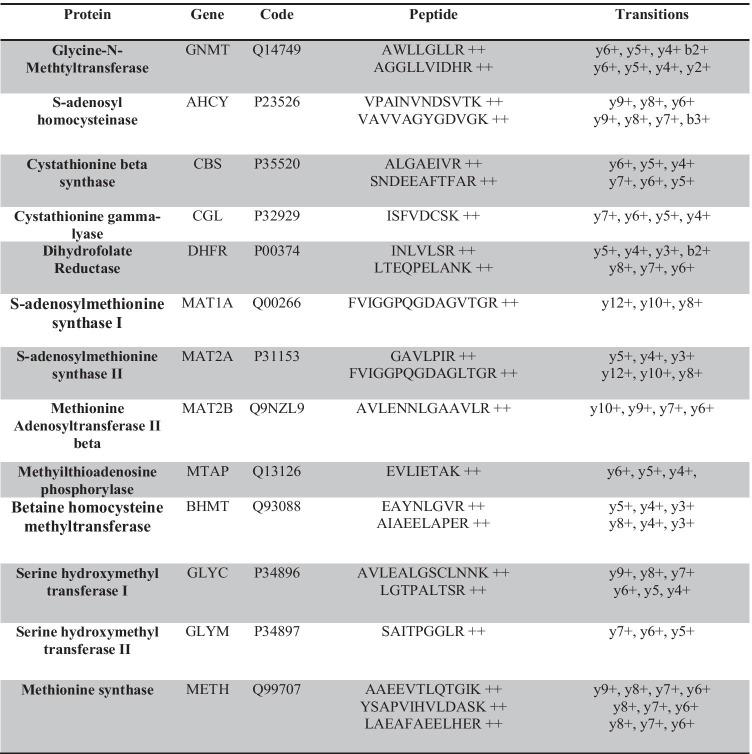
MRM transitions monitored to quantify one-carbon metabolism enzymes

## Supplementary Information

Below is the link to the electronic supplementary material.

Supplementary Table 1 (XLSX 5.42 MB)

Supplementary Table 2 (DOCX 15.3 KB)

Supplementary Table 3 (DOCX 17.2 KB)

Supplementary Figure 1 (PPTX 63 KB)

Supplementary Figure 2 (PPTX 629 KB)

Supplementary Figure 3 (DOCX 370 KB)

Supplementary Figure 4 (DOCX 1.24 KB)

Supplementary Figure 5 (PPTX 5.60 KB)

## Data Availability

Data are available for reviewers at Peptide Atlas: Server name: ftp.peptideatlas.org; Username: PASS01681; Password: NE4784q.

## Abbreviations

AHCY: Adenosylhomocysteinase
BHMT: Betaine-homocysteine S-methyltransferase
CBS: Cystationine beta-synthase
CGL: Cystationine gamma-lyase
DHFR: Dihydrofolate reductase
GNMT: Glycine N-methyltransferase
MAT1A: Methionine adenosyltransferasa 1A
MAT2A: Methionine adenosyltransferasa 2A
MAT2B: Methionine adenosyltransferasa 2B
MTAP: Methylthioadenosine phosphorylase
METH: Methionine synthase
SHMT1: Serine hydroxymethyltransferase 1
SHMT2: Serine hydroxymethyltransferase 2
