# Supplementary material for: Monitoring one-carbon metabolism by mass spectrometry to assess liver function and disease
Source: J Physiol Biochem. 2021 Dec 13;78(1):229–43. doi: 10.1007/s13105-021-00856-3 (PMC8666175; doi:10.1007/s13105-021-00856-3)
Supplement: Supplementary file 2 — Supplementary Table 2 (DOCX 15.3 KB) [file 13105_2021_856_MOESM2_ESM.docx]

**Supplementary table 2. Optimized collision energy values.** Collision energy (CE) parameter was optimized for each peptide to assure the maximum efficiency of precursor ion fragmentation at the collision cell. 250 fmol of each synthetic peptide spiked in 1 μg of digested proteome of *E. Coli* were injected in QTRAP5500. Several values of collision energy were applied for each peptide. The table shows the CE value that allowed the greatest intensity.

| **Protein** | **Peptide** | **Collision Energy optimized value** |
| --- | --- | --- |
| GNMT | AWLLGLLR | 22.1 |
|  | AGGLLVIDHR | 24.8 |
| AHCY | VAVVAGYGDVGK | 26.8 |
|  | VPAINVNDSVTK | 29.8 |
| CBS | ALGAEIVR | 19.3 |
|  | SNDEEAFTFAR | 30.5 |
| CGL | ISFVDCSK | 22.4 |
| DHFR | INLVLSR | 19.0 |
|  | LTEQPELANK | 27.0 |
| MAT1A | FVIGGPQGDAGVTGR | 34.1 |
| MAT2A | GAVLPIR | 16.8 |
|  | FVIGGPQGDAGLTGR | 34.4 |
| MAT2B | AVLENNLGAAVLR | 31.8 |
| MTAP | EVLIETAK | 21.1 |
|  | IGIIGGTGLDDPEILEGR | 27.2 |
| BHMT | EAYNLGVR | 21.6 |
|  | AIAEELAPER | 25.9 |
| SHMT1 | LGTPALTSR | 21.5 |
|  | AVLEALGSCLNNK | 33.0 |
| SHMT2 | SAITPGGLR | 20.4 |
| METH | YSAPVIHVLDASK | 33.3 |
|  | LAEAFAEELHER | 33.7 |
|  | AAEEVTLQTGIK | 29.9 |
