# Supplementary material for: Monitoring one-carbon metabolism by mass spectrometry to assess liver function and disease
Source: J Physiol Biochem. 2021 Dec 13;78(1):229–43. doi: 10.1007/s13105-021-00856-3 (PMC8666175; doi:10.1007/s13105-021-00856-3)
Supplement: Supplementary file 3 — Supplementary Table 3 (DOCX 17.2 KB) [file 13105_2021_856_MOESM3_ESM.docx]

**Supplementary table 3. Lower limit of detection (LLOD), lower limit of quantification (LLOQ) and upper limit of quantification (ULOQ).** LLOD, LLOQ and ULOQ were calculated for each peptide of the MRM method according to *Response curve* results. LLOD is defined as the blank signal*3. LLOQ is defined as the lower concentration with CV<20% across 3 replicates. ULOQ was defined as the maximum concentration at which the linearity is maintained. The linearity assay was performed removing one of the middle points of the curve and interpolating its concentration in the equation of the resulting curve. The calculated intensity must not exceed 5% of variation regarding the experimental value. Peptides marked in red did not showed linear behaviour. Peptides marked in orange did not pass subsequent stability assay experiment.

| Protein | Peptide | LLOD (fmol) | LLOQ (fmol) | ULOQ (fmol) |
| --- | --- | --- | --- | --- |
| GNMT | **VWQLYIGDTR** | 0.5 | 0.5 | 1000 |
|  | **AWLLGLLR** | 0.5 | 0.5 | 1000 |
|  | **AGGLLVIDHR** | 1 | 0.5 | 1000 |
| AHCY | **VADIGLAAWGR** | 0.5 | 1 | 1000 |
|  | **VPAINVNDSVTK** | 1 | 0.5 | 1000 |
|  | **VAVVAGYGDVGK** | 1 | 0.5 | 250 |
| CBS | **ILPDILK** | 0.5 | 0.5 | 125 |
|  | **ALGAEIVR** | 2 | 0.5 | 250 |
|  | **SNDEEAFTFAR** | 0.5 | 0.5 | 1000 |
| CGL | **ISFVDCSK** | 0.5 | 4 | 1000 |
|  | **LLEAAITPETK** | 0.5 | 1 | 1000 |
| DHFR | **NGDLPWPPLR** | 1 | 0.5 | 1000 |
|  | **QNLVIMGK** | 4 | 2 | 1000 |
|  | **INLVLSR** | 2 | 0.5 | 1000 |
|  | **LTEQPELANK** | 1 | 0.5 | 500 |
| MAT1A | **SGLLPWLRPDSK** | 0.5 | 0.5 | 1000 |
|  | **FVIGGPQGDAGVTGR** | 1 | 0.5 | 1000 |
| MAT2A | **GAVLPIR** | 8 | 0.5 | 250 |
|  | **FVIGGPQGDAGLTGR** | 2 | 0.5 | 1000 |
|  | **TAAYGHFGR** | 16 | NO LINEAL | - |
| MAT2B | **VLVTGATGLLGR** | 8 | 1 | 1000 |
|  | **AVLENNLGAAVLR** | 0.5 | 0.5 | 1000 |
| MTAP | **IGIIGGTGLDDPEILEGR** | 0.5 | 0.5 | 500 |
|  | **EVLIETAK** | 4 | 0.5 | 500 |
|  | **AESFMFR** | 0.5 | 0.5 | 1000 |
| BHMT | **GNYVLEK** | 4 | NO LINEAL | - |
|  | **ISGQEVNEAACDIAR** | 1 | 0.5 | 1000 |
|  | **EAYNLGVR** | 1 | 0.5 | 500 |
|  | **AIAEELAPER** | 2 | 0.5 | 1000 |
| SHMT1 | **VGLELIASENFASR** | 0.5 | 0.5 | 500 |
|  | **AVLEALGSCLNNK** | 0.5 | 0.5 | 100 |
|  | **LGTPALTSR** | 4 | 0.5 | 1000 |
| SHMT2 | **TGLIDYNQLALTAR** | 1 | 0.5 | 1000 |
|  | **EYSLQVLK** | 2 | 0.5 | 1000 |
|  | **SAITPGGLR** | 2 | 0.5 | 500 |
| METH | **AAEEVTLQTGIK** | 1 | 0.5 | 1000 |
|  | **IPLLIGGATTSK** | 1 | 0.5 | 1000 |
|  | **YSAPVIHVLDASK** | 2 | 1 | 1000 |
|  | **LAEAFAEELHER** | 1 | 0.5 | 1000 |

• Validated peptides

• Non validated peptides: CVt > 20%

• Non validated peptides: no lineality
