# Supplementary material for: Monitoring one-carbon metabolism by mass spectrometry to assess liver function and disease
Source: J Physiol Biochem. 2021 Dec 13;78(1):229–43. doi: 10.1007/s13105-021-00856-3 (PMC8666175; doi:10.1007/s13105-021-00856-3)
Supplement: Supplementary file 4 — Supplementary Figure 1 (PPTX 63 KB) [file 13105_2021_856_MOESM4_ESM.pptx]

## Slide 1
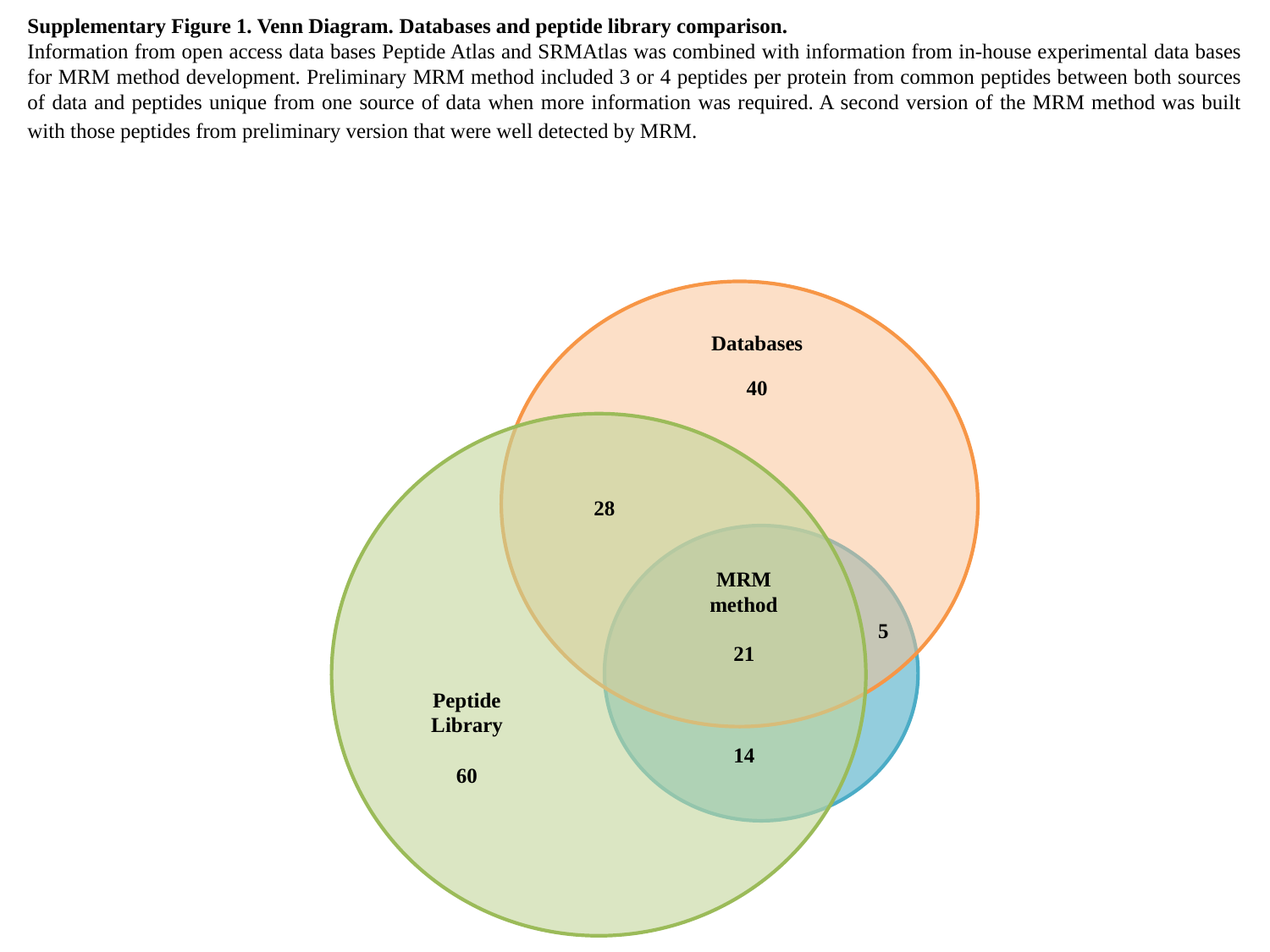

Supplementary Figure 1. Venn Diagram. Databases and peptide library comparison.
Information from open access data bases Peptide Atlas and SRMAtlas was combined with information from in-house experimental data bases for MRM method development. Preliminary MRM method included 3 or 4 peptides per protein from common peptides between both sources of data and peptides unique from one source of data when more information was required. A second version of the MRM method was built with those peptides from preliminary version that were well detected by MRM.
Databases
40
28
MRM method
5
21
Peptide Library
14
60
