# Supplementary material for: Monitoring one-carbon metabolism by mass spectrometry to assess liver function and disease
Source: J Physiol Biochem. 2021 Dec 13;78(1):229–43. doi: 10.1007/s13105-021-00856-3 (PMC8666175; doi:10.1007/s13105-021-00856-3)
Supplement: Supplementary file 5 — Supplementary Figure 2 (PPTX 629 KB) [file 13105_2021_856_MOESM5_ESM.pptx]

## Slide 1
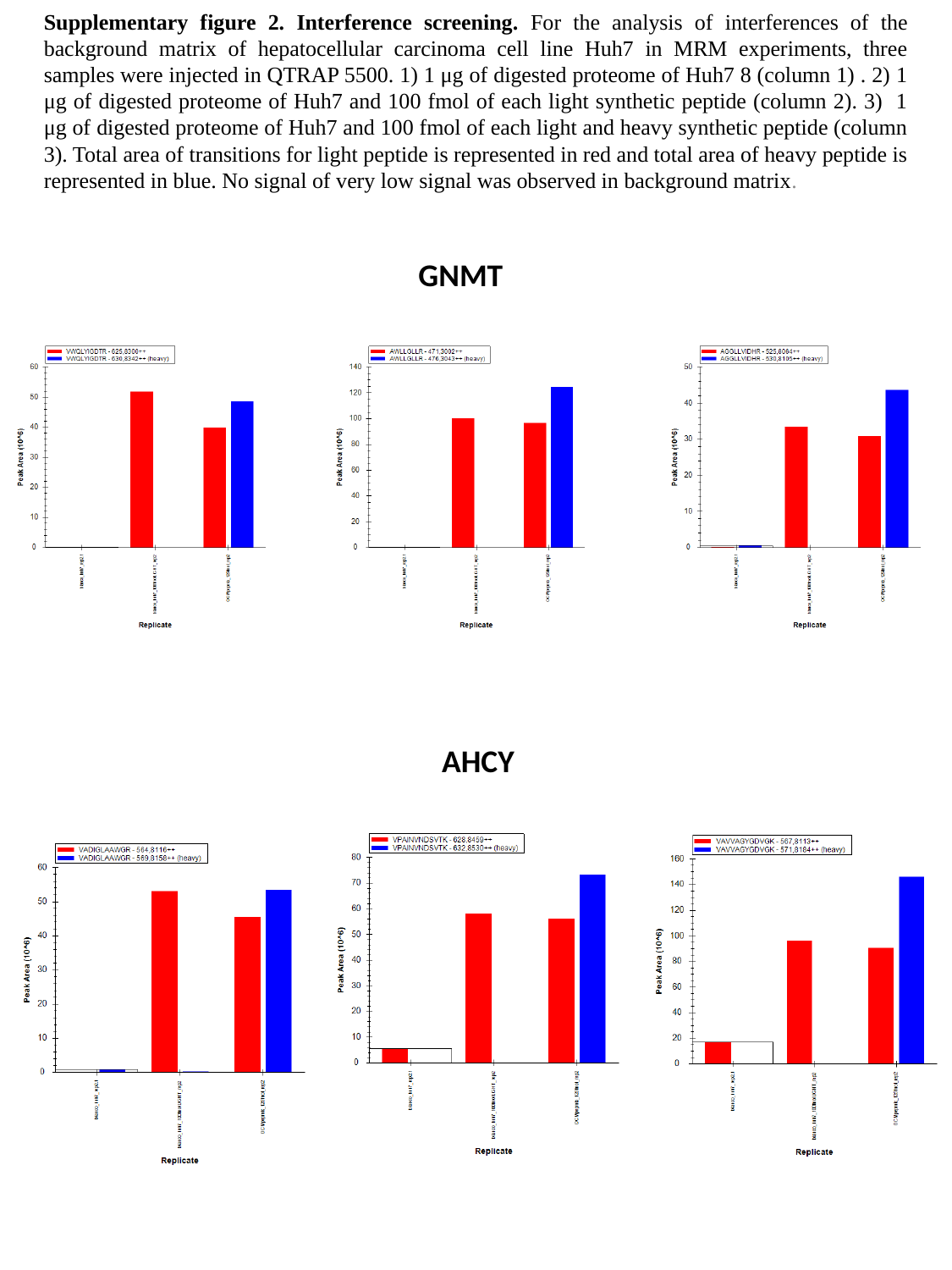

Supplementary figure 2. Interference screening. For the analysis of interferences of the background matrix of hepatocellular carcinoma cell line Huh7 in MRM experiments, three samples were injected in QTRAP 5500. 1) 1 μg of digested proteome of Huh7 8 (column 1) . 2) 1 μg of digested proteome of Huh7 and 100 fmol of each light synthetic peptide (column 2). 3) 1 μg of digested proteome of Huh7 and 100 fmol of each light and heavy synthetic peptide (column 3). Total area of transitions for light peptide is represented in red and total area of heavy peptide is represented in blue. No signal of very low signal was observed in background matrix.
GNMT
AHCY

## Slide 2
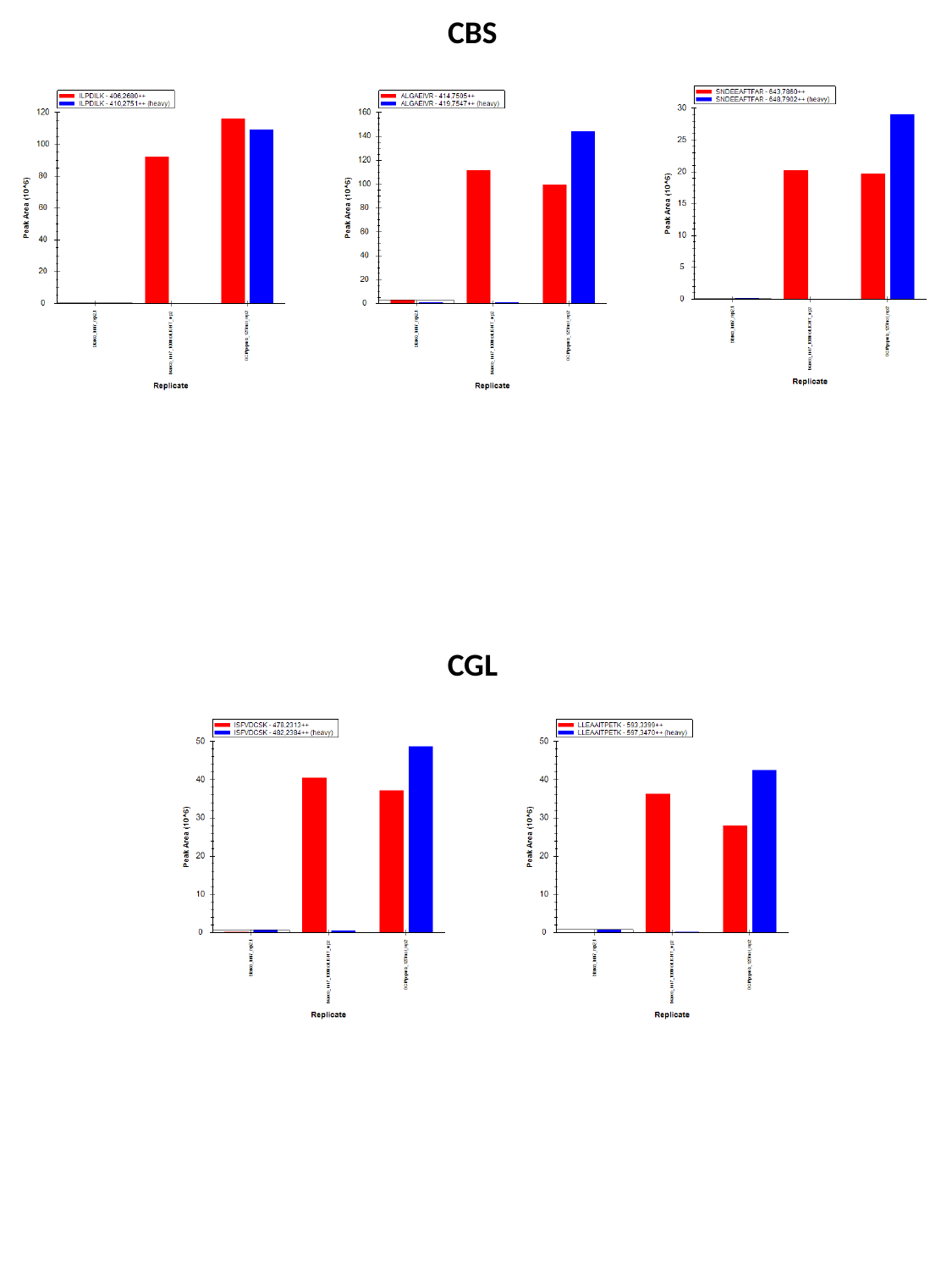

CBS
CGL

## Slide 3
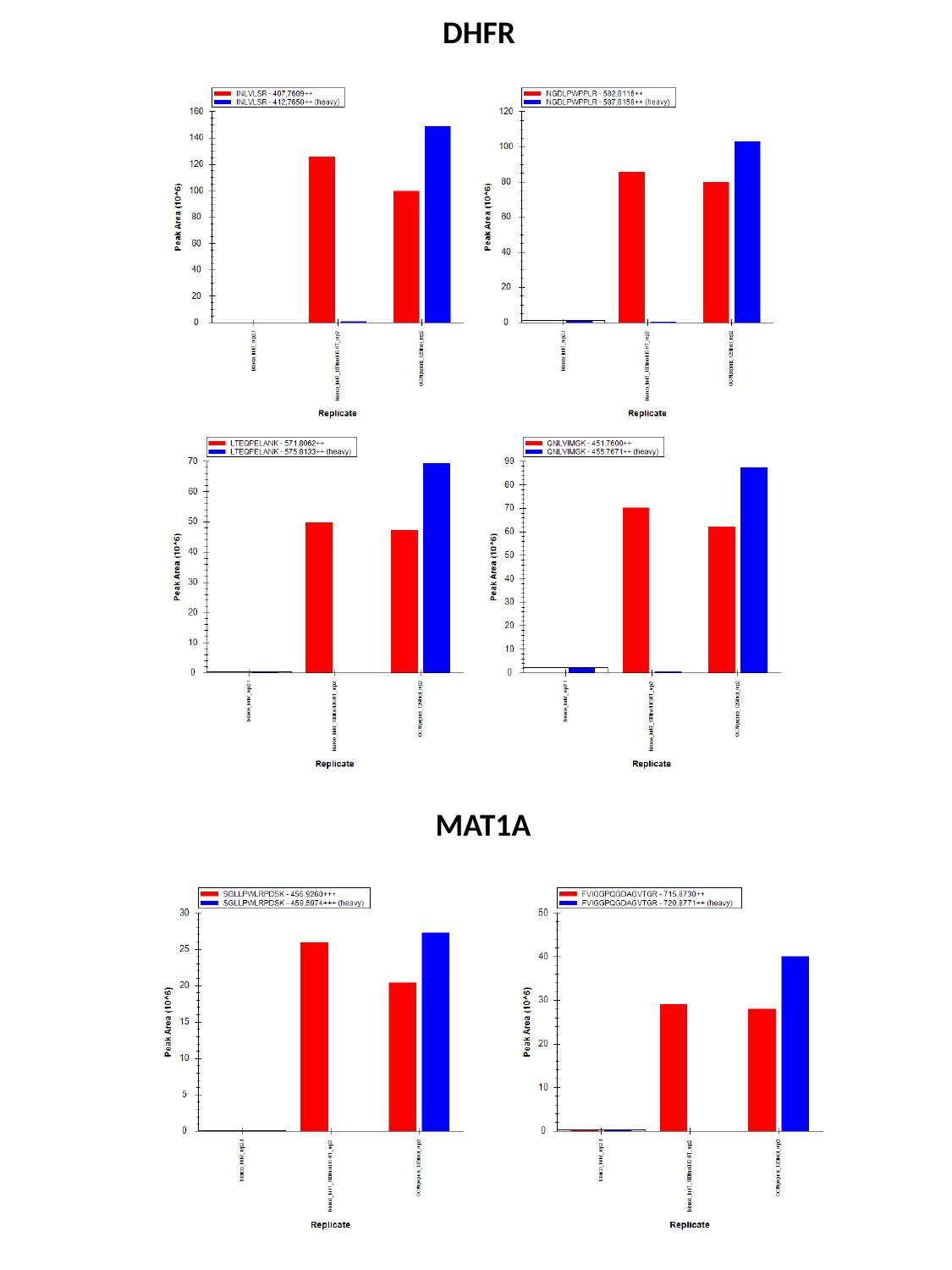

DHFR
MAT1A

## Slide 4
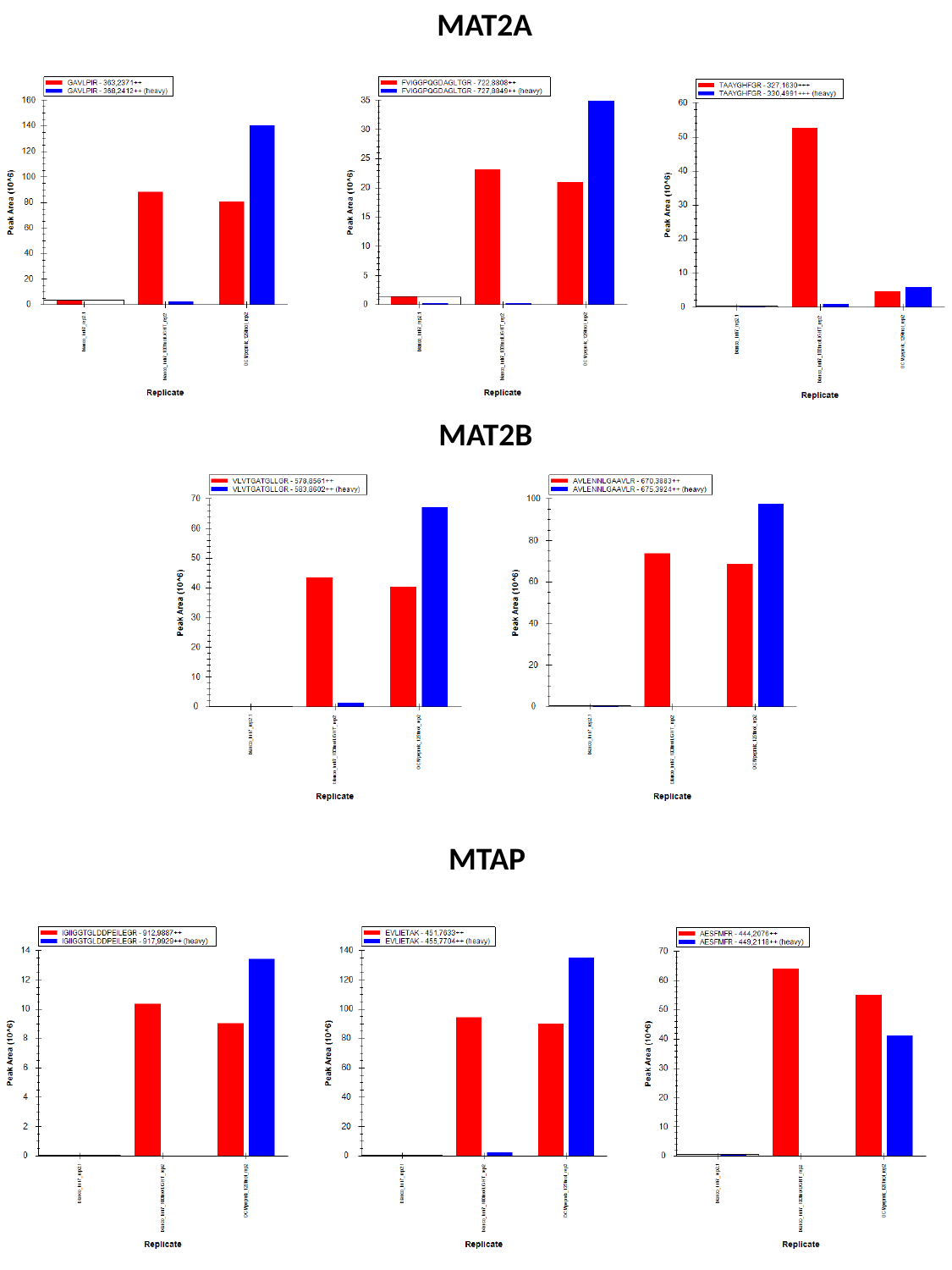

MAT2A
MAT2B
MTAP

## Slide 5
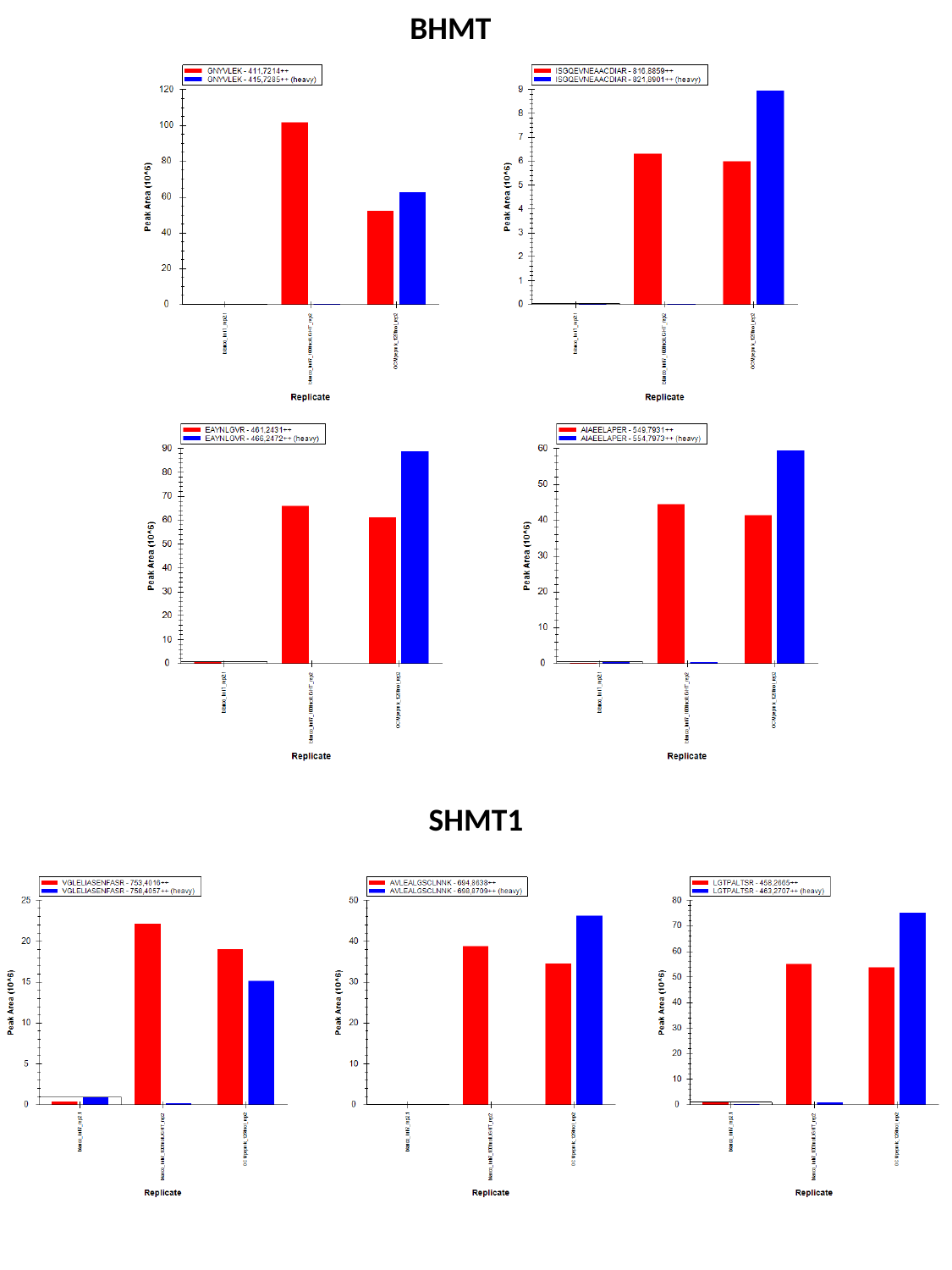

BHMT
SHMT1

## Slide 6
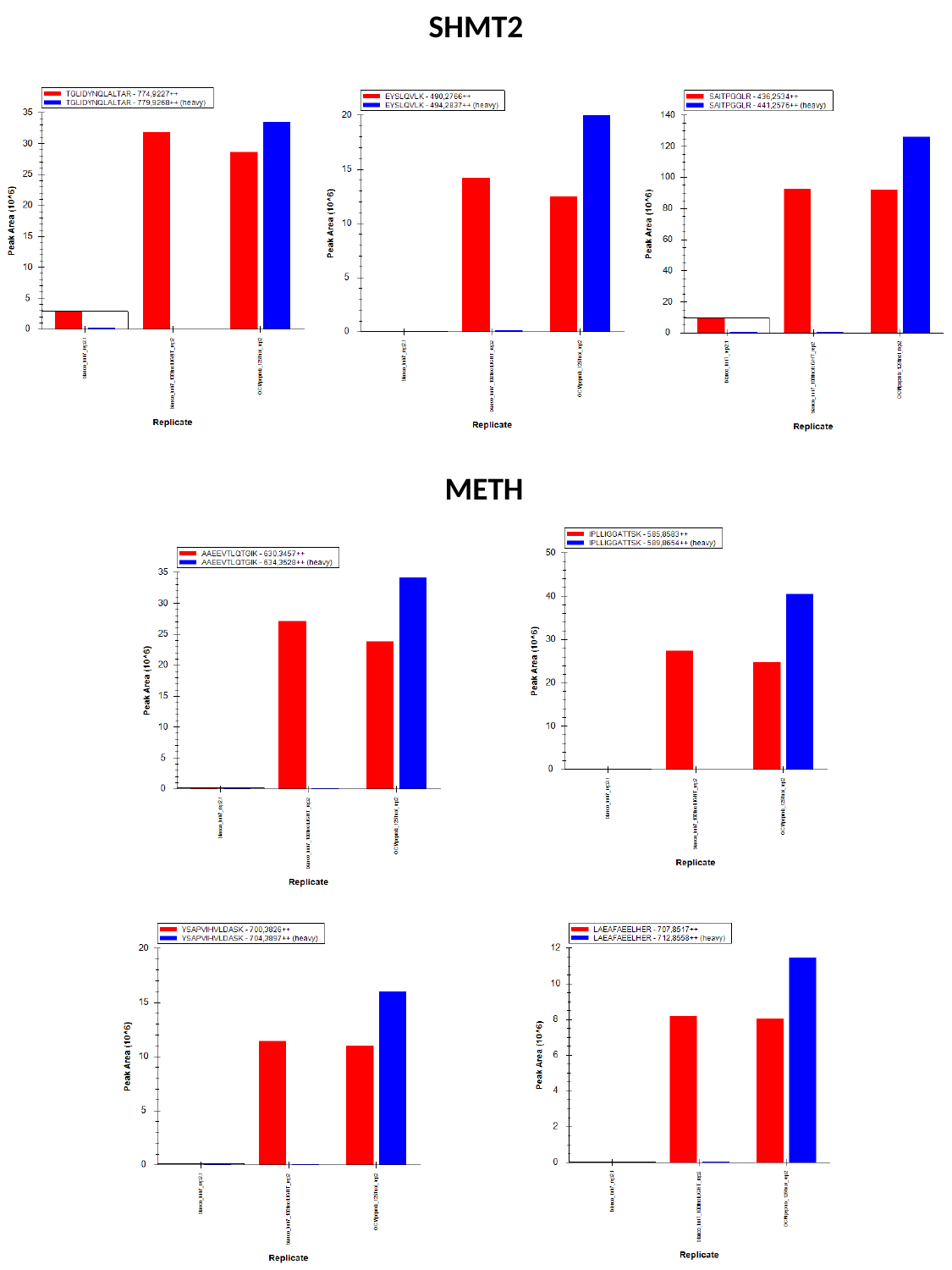

SHMT2
METH
