# Supplementary material for: Monitoring one-carbon metabolism by mass spectrometry to assess liver function and disease
Source: J Physiol Biochem. 2021 Dec 13;78(1):229–43. doi: 10.1007/s13105-021-00856-3 (PMC8666175; doi:10.1007/s13105-021-00856-3)
Supplement: Supplementary file 6 — Supplementary Figure 3 (DOCX 370 KB) [file 13105_2021_856_MOESM6_ESM.docx]

**Supplementary figure 3. Response curve.** For each peptide of the MRM method, 12 concentration dilution points of heavy labelled synthetic peptide were injected from 0,5 fmol to 1pmol. All injections included 1 μg of digested proteome of Huh7 and 100 fmol of light synthetic peptides

|  | **AWLLGLLR** | **AGGLLVIDHR** | **VWQLYIGDTR** |
| --- | --- | --- | --- |
| Slope | 0,009246 | 0,01173 | 0,01150 |
| Y-intercept | 0,05601 | -0,01368 | -0,08580 |
| X-intercept | -6,057 | 1,167 | 7,460 |
| **R square** | **0,9889** | **0,9992** | **0,9896** |
|  |  |  |  |

|  | **VPAINVNDSVTK** | **VAVVAGYGDVGK** | **VADIGLAAWGR** |
| --- | --- | --- | --- |
|  |  |  |  |
| Slope | 0,009800 | 0,01089 | 0,009843 |
| Y-intercept | 0,02461 | 0,08834 | -0,02612 |
| X-intercept | -2,512 | -8,112 | 2,654 |
| **R square** | **0,9983** | **0,9913** | **0,9929** |

|  | **ALGAEIVR** | **SNDEEAFTFAR** | **ILPDILK** |
| --- | --- | --- | --- |
| Slope | 0,005756 | 0,01202 | 0,001908 |
| Y-intercept | 0,2617 | -0,002633 | 0,2256 |
| X-intercept | -45,47 | 0,2190 | -118,2 |
| **R square** | **0,9395** | **0,9991** | **0,7544** |
|  |  |  |  |

|  | **LLEAAITPETK** | **ISFVDCSK** |
| --- | --- | --- |
| Slope | 0,01224 | 0,01058 |
| Y-intercept | 0,009776 | 0,008235 |
| X-intercept | -0,7990 | -0,7781 |
| **R square** | **0,9984** | **0,9978** |

|  | **NGDLPWPPLR** | **QNLVIMGK** | **INLVLSR** | **LTEQPELANK** |
| --- | --- | --- | --- | --- |
| Slope | 0,009147 | 0,01042 | 0,009064 | 0,008809 |
| Y-intercept | 0,05520 | 0,03520 | 0,1160 | 0,1246 |
| X-intercept | -6,034 | -3,379 | -12,80 | -14,15 |
| **R square** | **0,9873** | **0,9961** | **0,9918** | **0,9917** |
|  |  |  |  |  |

|  | **FVIGGPQGDAGVTGR** | **SGLLPWLRPDSK** |
| --- | --- | --- |
| Slope | 0,01125 | 0,01150 |
| Y-intercept | 0,01783 | -0,03661 |
| X-intercept | -1,585 | 3,183 |
| **R square** | **0,9995** | **0,9957** |
|  |  |  |

|  | **GAVLPIR** | **FVIGGPQGDAGLTGR** | **TAAYGHFGR** |
| --- | --- | --- | --- |
| Slope | 0,008688 | 0,01289 | 0,008289 |
| Y-intercept | 0,2578 | 0,02033 | 0,1846 |
| X-intercept | -29,68 | -1,577 | -22,27 |
| **R square** | **0,9682** | **0,9993** | **0,9682** |

|  | **AVLENNLGAAVLR** | **VLVTGATGLLGR** |
| --- | --- | --- |
| Slope | 0,01014 | 0,01294 |
| Y-intercept | 0,05013 | 0,03042 |
| X-intercept | -4,943 | -2,351 |
| **R square** | **0,9983** | **0,9953** |

|  | **IGIIGGTGLDDPEILEGR** | **EVLIETAK** | **AESFMFR** |
| --- | --- | --- | --- |
| Slope | 0,01470 | 0,008850 | 0,005527 |
| Y-intercept | -0,1496 | 0,1510 | 0,01377 |
| X-intercept | 10,18 | -17,06 | -2,491 |
| **R square** | **0,9926** | **0,9899** | **0,9924** |

|  | **EAYNLGVR** | **AIAEELAPER** | **GNYVLEK** | **ISGQEVNEAACDIAR** |
| --- | --- | --- | --- | --- |
| Slope | 0,008939 | 0,01101 | 0,008130 | 0,01258 |
| Y-intercept | 0,1116 | 0,03897 | 0,09180 | -0,02816 |
| X-intercept | -12,49 | -3,540 | -11,29 | 2,238 |
| **R square** | **0,9894** | **0,9955** | **0,9664** | **0,9995** |
|  |  |  |  |  |

|  | **AVLEALGSCLNNK** | **LGTPALTSR** | **VGLELIASENFASR** |
| --- | --- | --- | --- |
| Slope | 0,009558 | 0,009020 | 0,01102 |
| Y-intercept | 0,05421 | 0,1037 | -0,2323 |
| X-intercept | -5,672 | -11,50 | 21,07 |
| **R square** | **0,9980** | **0,9946** | **0,9668** |
|  |  |  |  |

|  | **TGLIDYNQLALTAR** | **SAITPGGLR** | **EYSLQVLK** |
| --- | --- | --- | --- |
| Slope | 0,009893 | 0,007312 | 0,01379 |
| Y-intercept | -0,03249 | 0,1677 | -0,04014 |
| X-intercept | 3,284 | -22,94 | 2,910 |
| **R square** | **0,9988** | **0,9793** | **0,9968** |
|  |  |  |  |

|  | **AAEEVTLQTGIK** | **YSAPVIHVLDASK** | **LAEAFAEELHER** | **IPLLIGGATTSK** |
| --- | --- | --- | --- | --- |
| Slope | 0,01240 | 0,01209 | 0,01238 | 0,01360 |
| Y-intercept | -0,04392 | -0,02059 | -0,03315 | -0,02121 |
| X-intercept | 3,541 | 1,703 | 2,678 | 1,559 |
| **R square** | **0,9978** | **0,9999** | **0,9996** | **0,9964** |
