# Supplementary material for: Monitoring one-carbon metabolism by mass spectrometry to assess liver function and disease
Source: J Physiol Biochem. 2021 Dec 13;78(1):229–43. doi: 10.1007/s13105-021-00856-3 (PMC8666175; doi:10.1007/s13105-021-00856-3)
Supplement: Supplementary file 7 — Supplementary Figure 4 (DOCX 1.24 KB) [file 13105_2021_856_MOESM7_ESM.docx]

**Supplementary figure 4. Minivalidation of repeatability assay.** 3 replicates of 3 concentrations prepared in different days were analysed in 5 different days on a QTRAP 5500. Low concentration included 5 fmol of heavy synthetic peptide and 30 fmol of light synthetic peptide, medium concentration included 50 fmol of heavy synthetic peptide and 30 fmol of light synthetic peptide and high concentration included 500 fmol of heavy synthetic peptide and 30 fmol of light synthetic peptide. All injections included 1 μg of Huh7 digested proteome.
