# Supplementary material for: Monitoring one-carbon metabolism by mass spectrometry to assess liver function and disease
Source: J Physiol Biochem. 2021 Dec 13;78(1):229–43. doi: 10.1007/s13105-021-00856-3 (PMC8666175; doi:10.1007/s13105-021-00856-3)
Supplement: Supplementary file 8 — Supplementary Figure 5 (PPTX 5.60 KB) [file 13105_2021_856_MOESM8_ESM.pptx]

## Slide 1
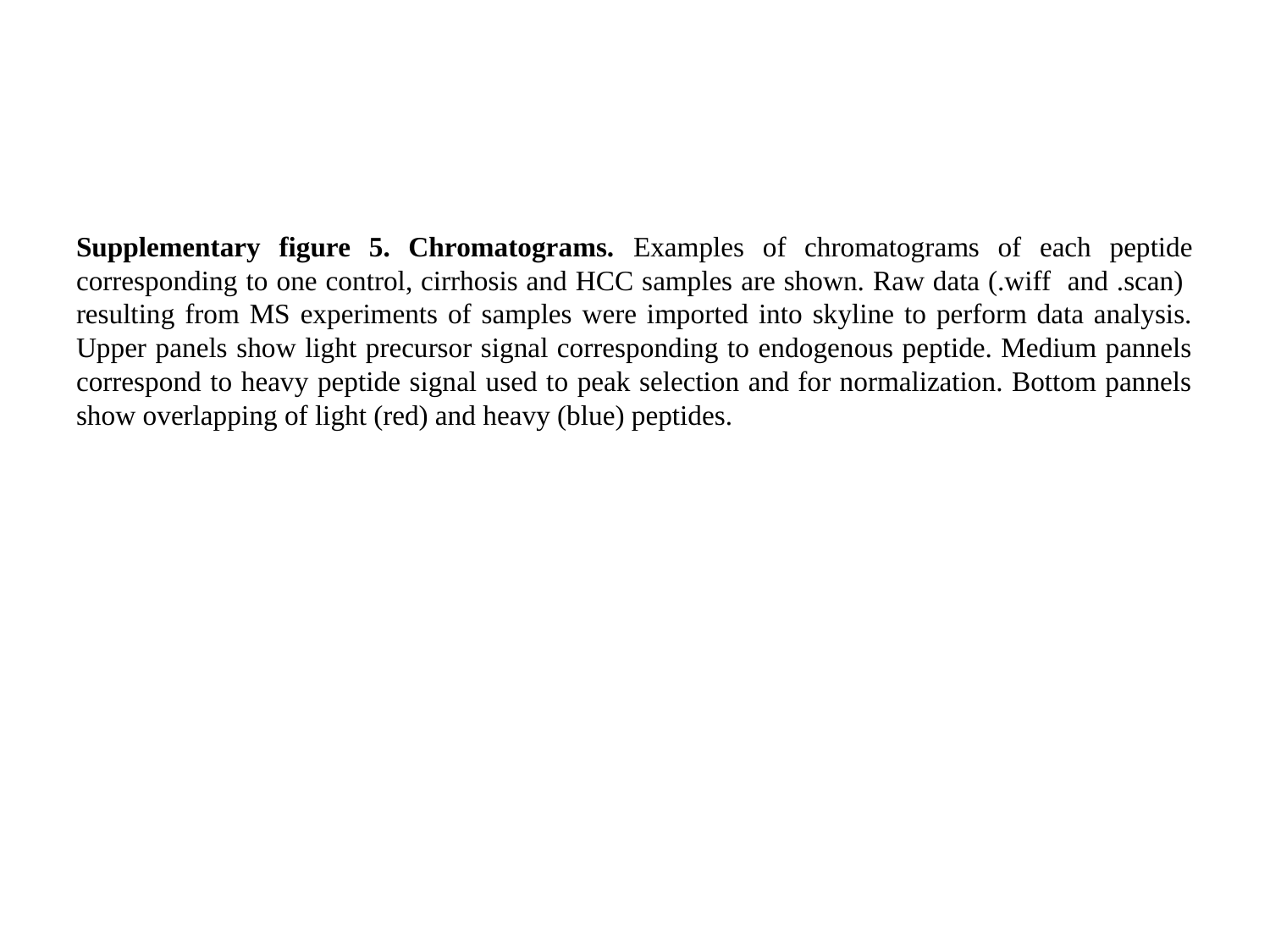

Supplementary figure 5. Chromatograms. Examples of chromatograms of each peptide corresponding to one control, cirrhosis and HCC samples are shown. Raw data (.wiff and .scan) resulting from MS experiments of samples were imported into skyline to perform data analysis. Upper panels show light precursor signal corresponding to endogenous peptide. Medium pannels correspond to heavy peptide signal used to peak selection and for normalization. Bottom pannels show overlapping of light (red) and heavy (blue) peptides.

## Slide 2
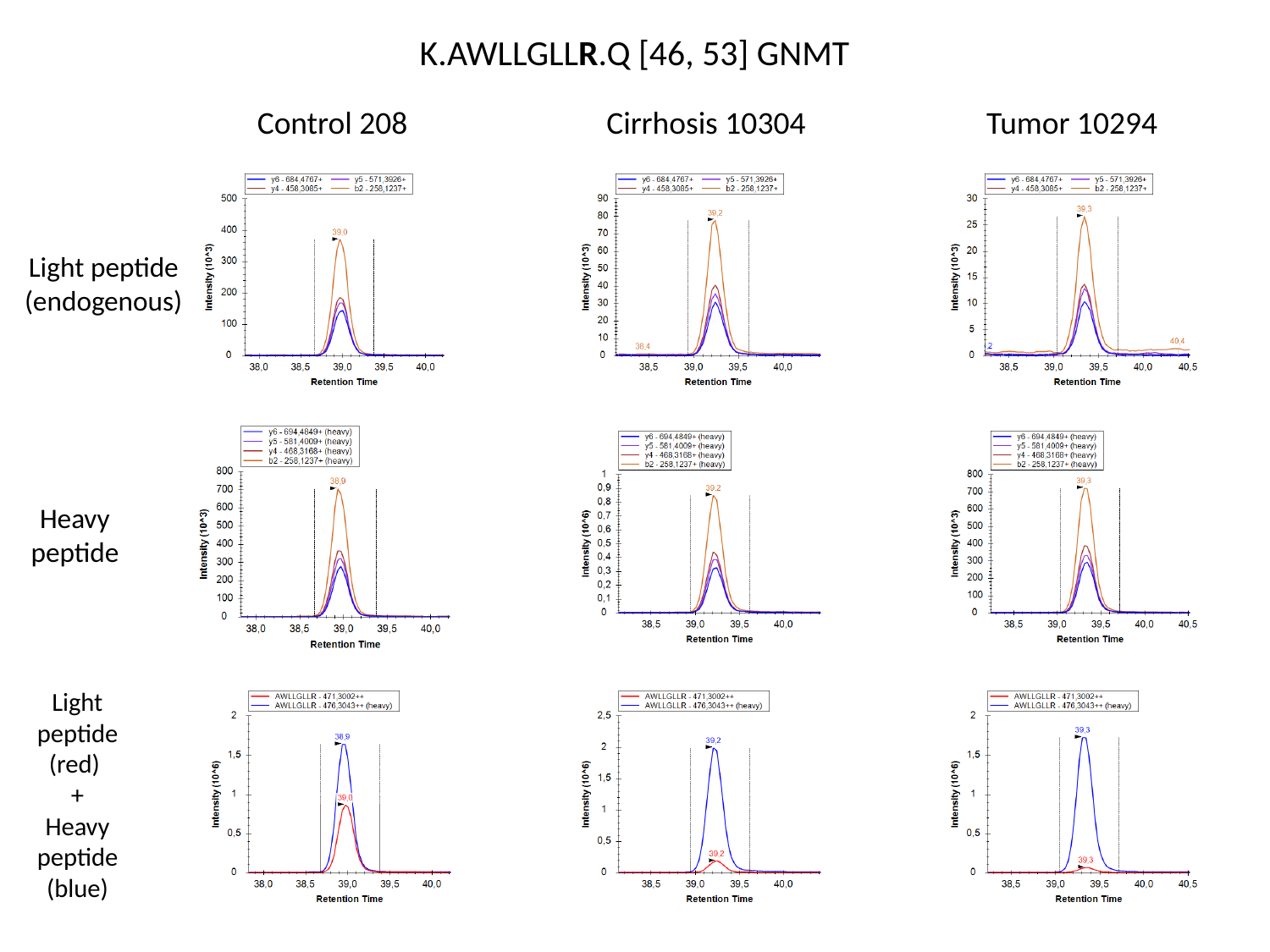

K.AWLLGLLR.Q [46, 53] GNMT
Control 208
Cirrhosis 10304
Tumor 10294
Light peptide
(endogenous)
Heavy peptide
Light peptide (red)
+
Heavy peptide (blue)

## Slide 3
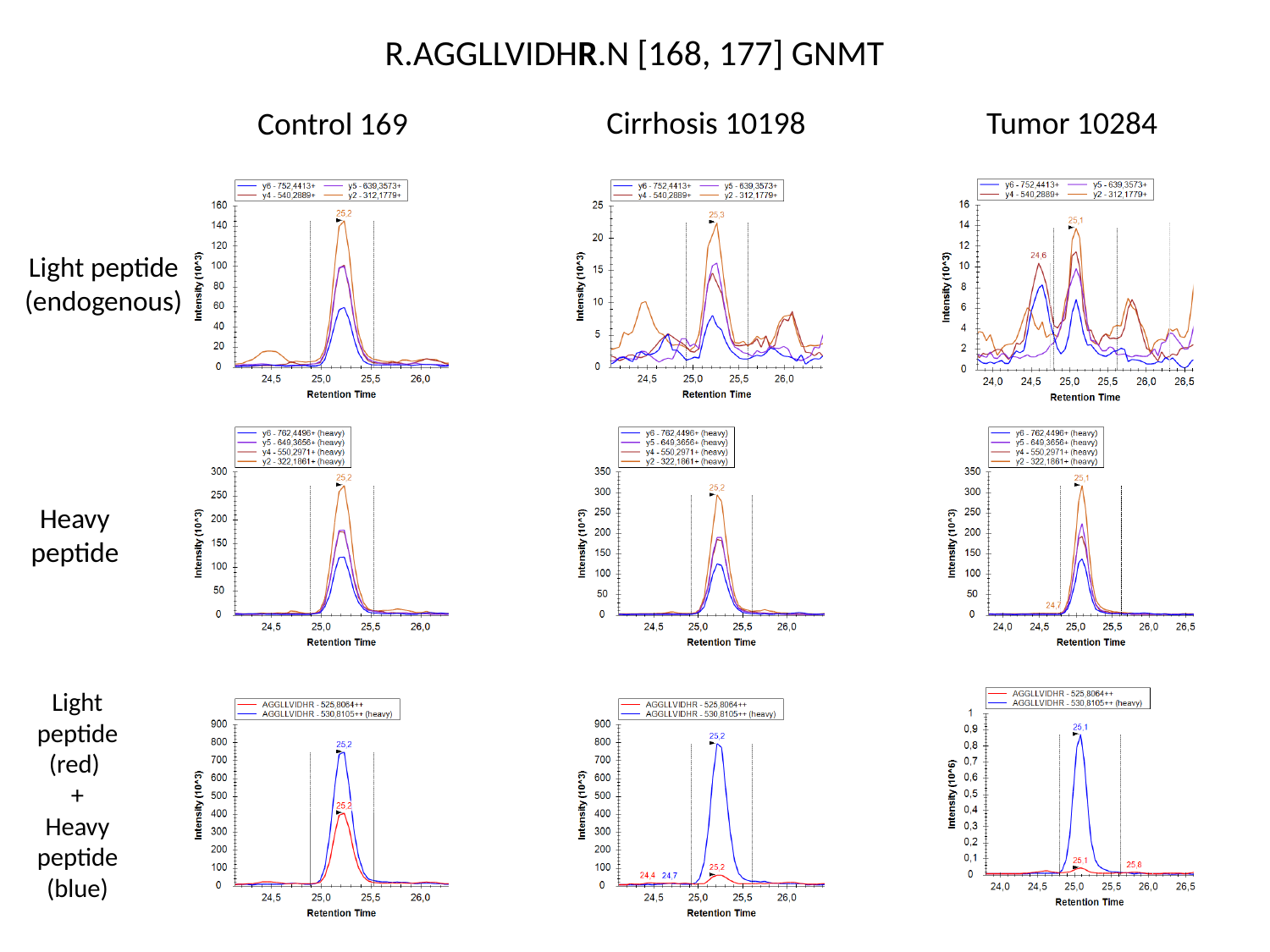

# R.AGGLLVIDHR.N [168, 177] GNMT
Control 169
Cirrhosis 10198
Tumor 10284
Light peptide
(endogenous)
Heavy peptide
Light peptide (red)
+
Heavy peptide (blue)

## Slide 4
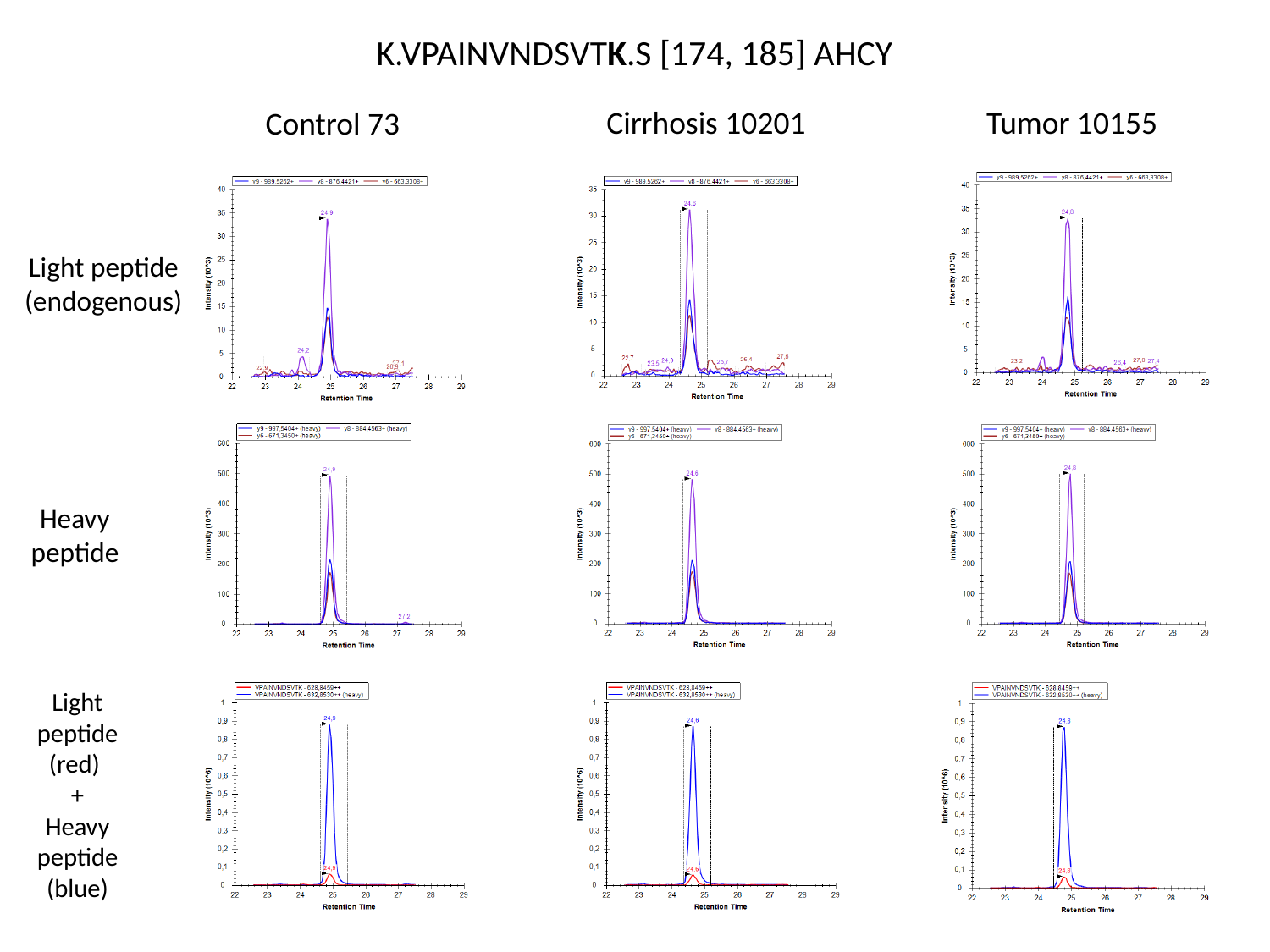

K.VPAINVNDSVTK.S [174, 185] AHCY
Control 73
Cirrhosis 10201
Tumor 10155
Light peptide
(endogenous)
Heavy peptide
Light peptide (red)
+
Heavy peptide (blue)

## Slide 5
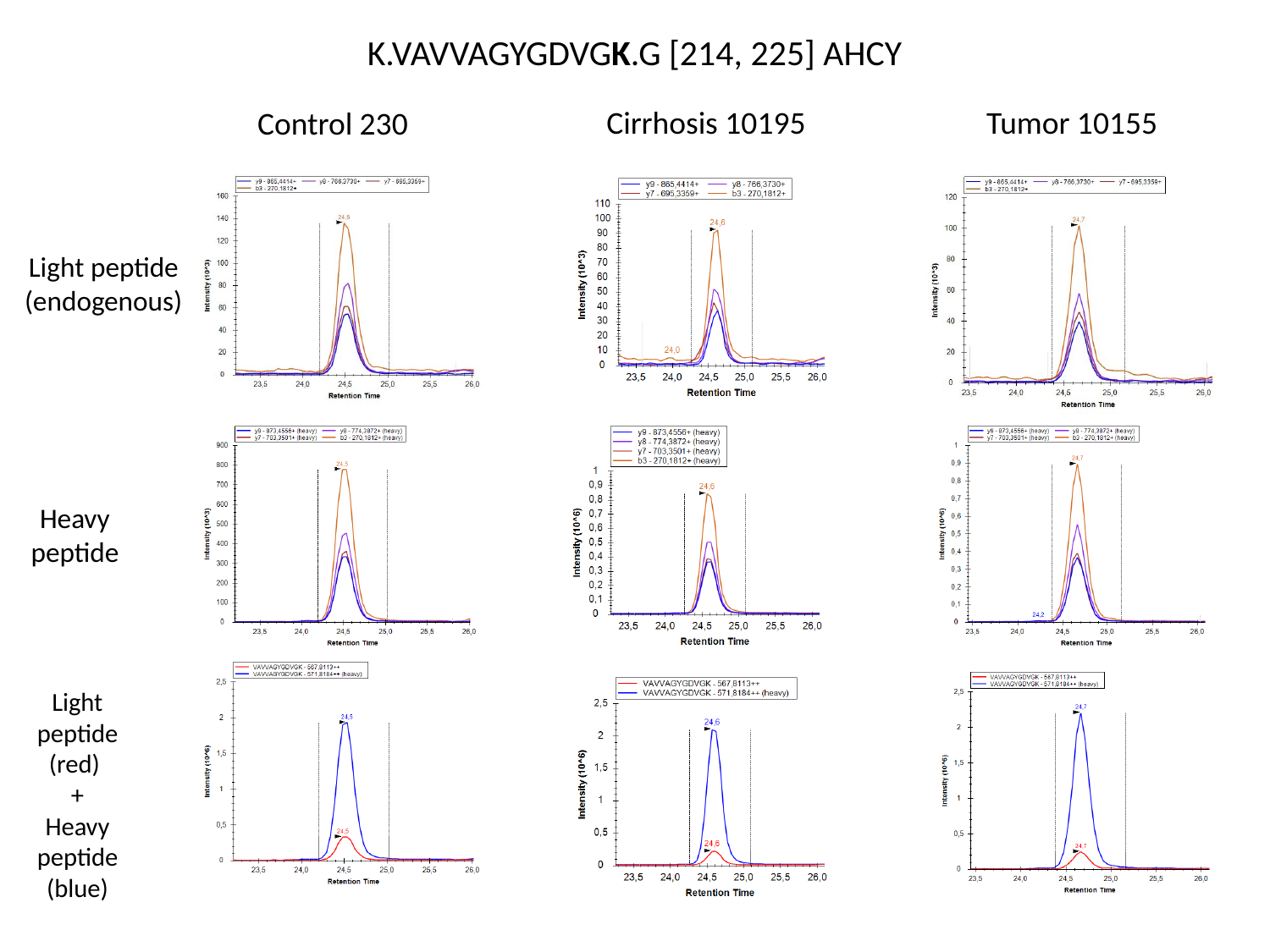

K.VAVVAGYGDVGK.G [214, 225] AHCY
Control 230
Cirrhosis 10195
Tumor 10155
Light peptide
(endogenous)
Heavy peptide
Light peptide (red)
+
Heavy peptide (blue)

## Slide 6
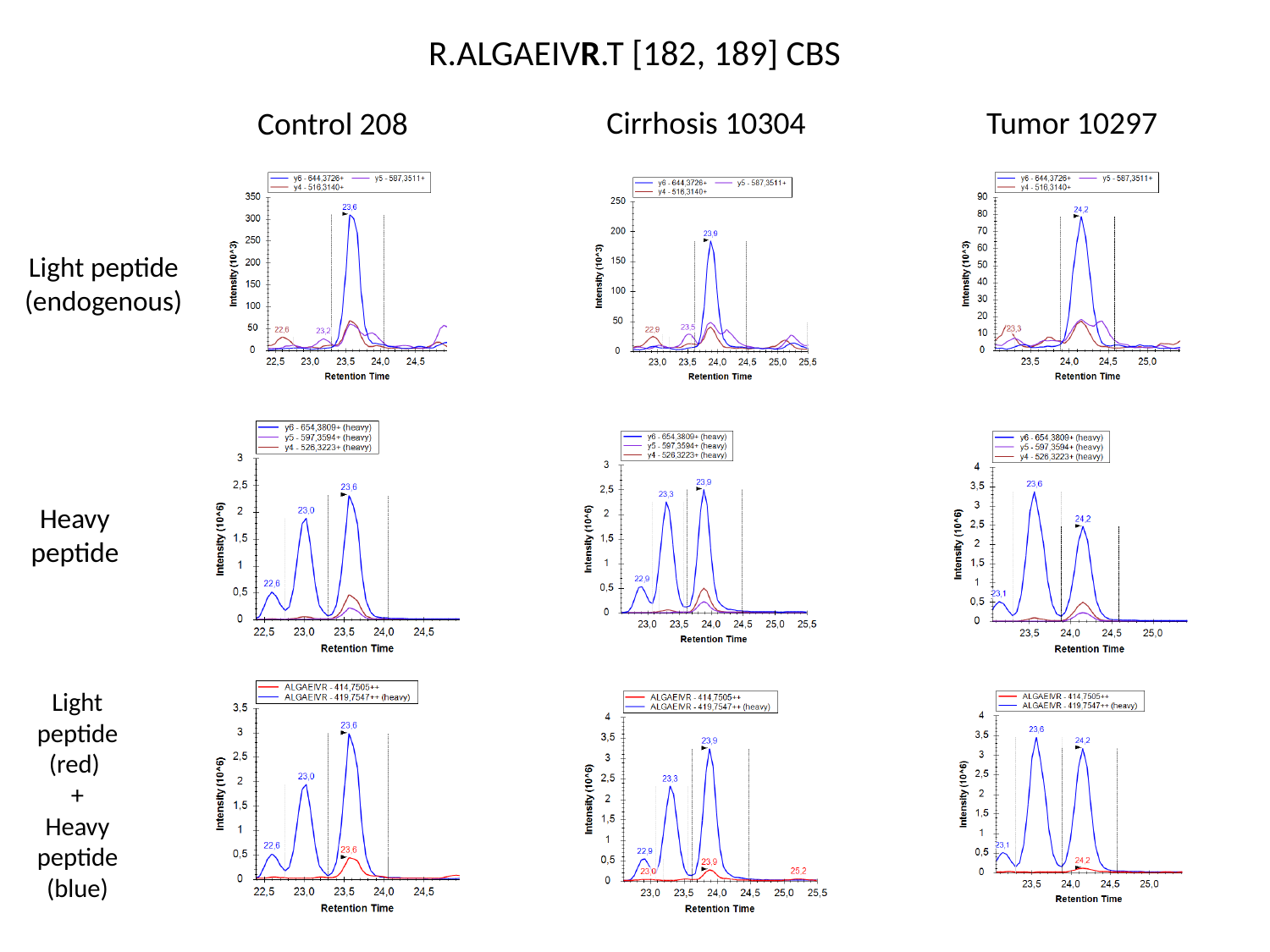

R.ALGAEIVR.T [182, 189] CBS
Control 208
Cirrhosis 10304
Tumor 10297
Light peptide
(endogenous)
Heavy peptide
Light peptide (red)
+
Heavy peptide (blue)

## Slide 7
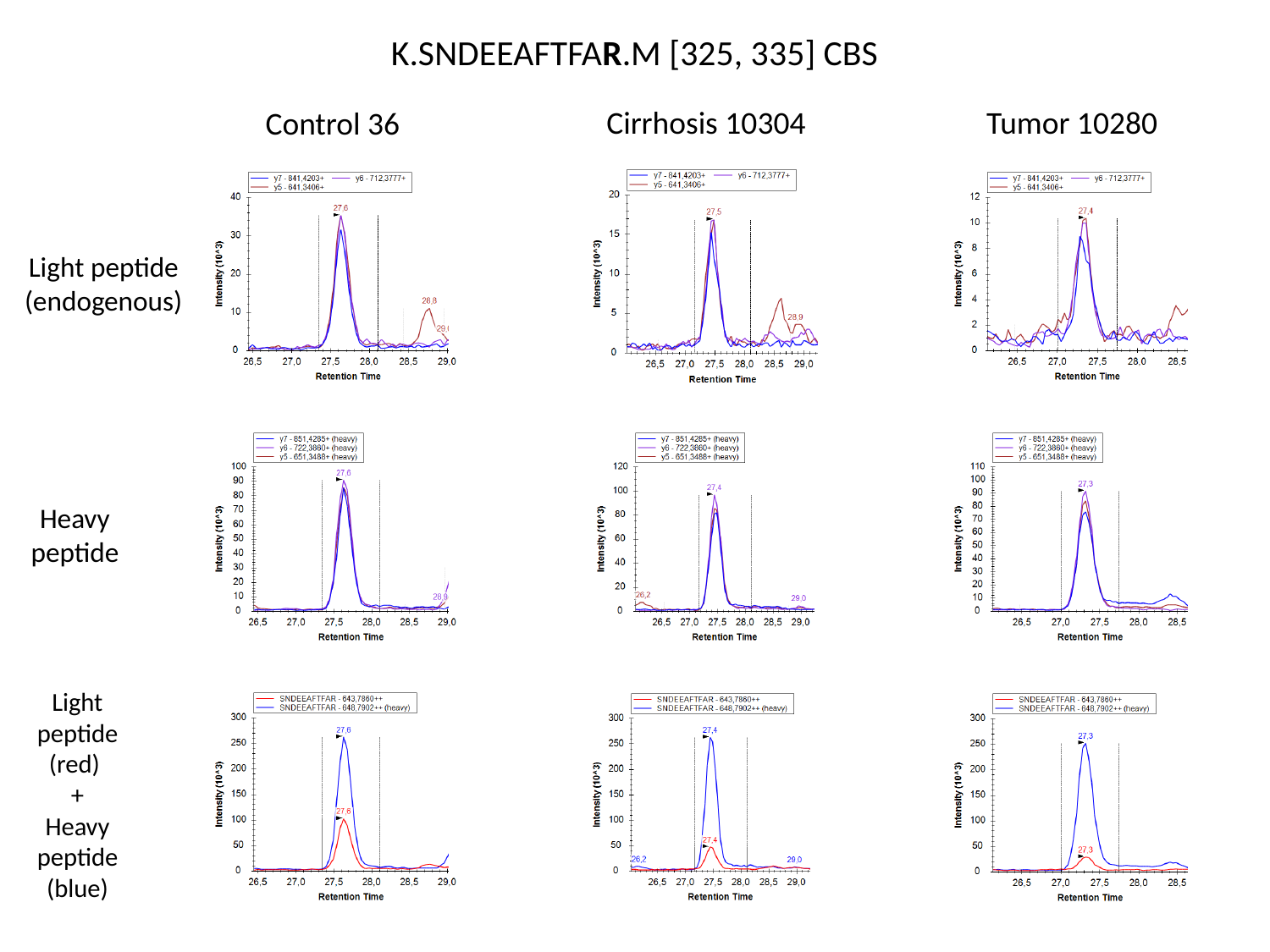

K.SNDEEAFTFAR.M [325, 335] CBS
Control 36
Cirrhosis 10304
Tumor 10280
Light peptide
(endogenous)
Heavy peptide
Light peptide (red)
+
Heavy peptide (blue)

## Slide 8
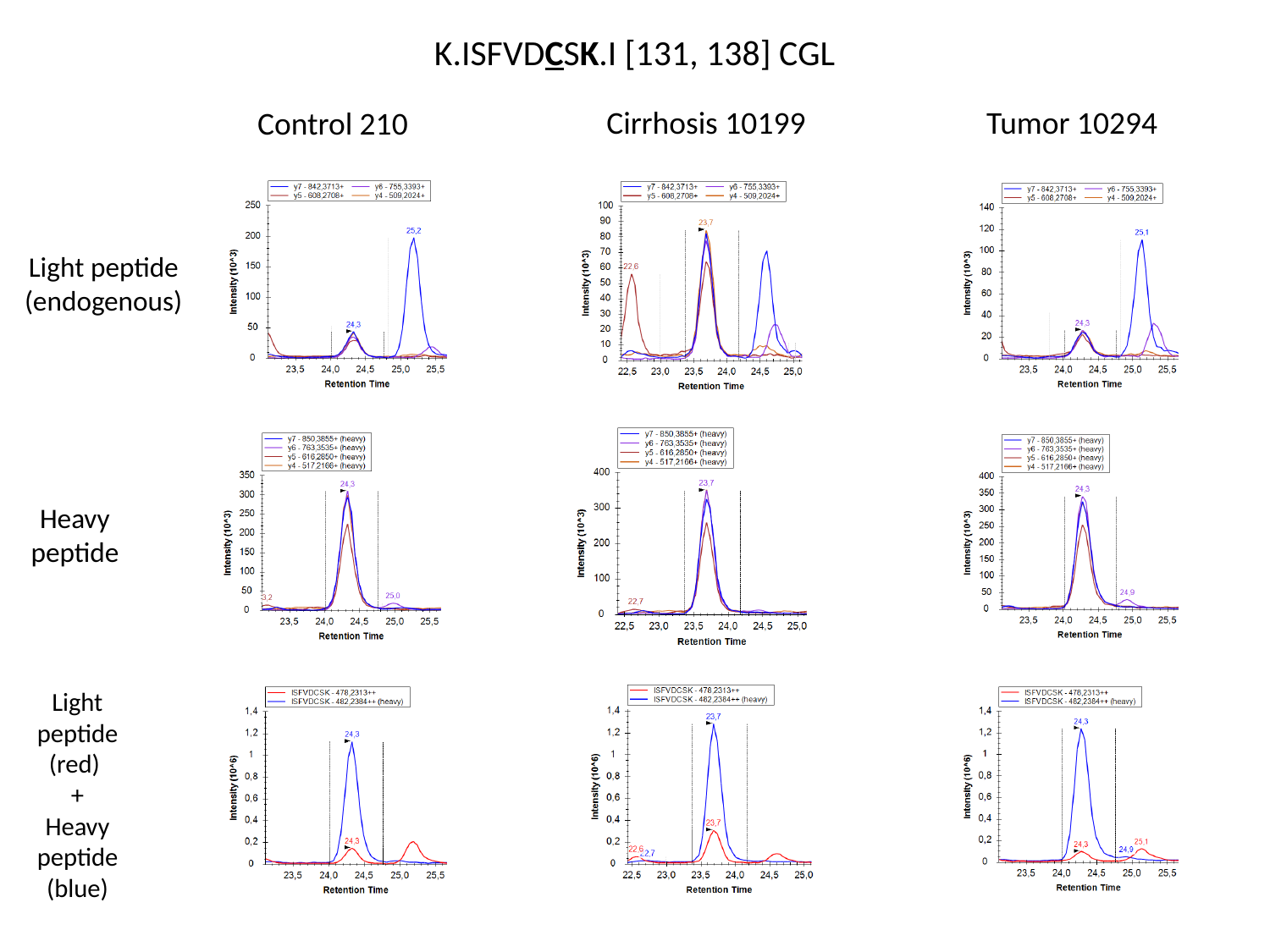

K.ISFVDCSK.I [131, 138] CGL
Control 210
Cirrhosis 10199
Tumor 10294
Light peptide
(endogenous)
Heavy peptide
Light peptide (red)
+
Heavy peptide (blue)

## Slide 9
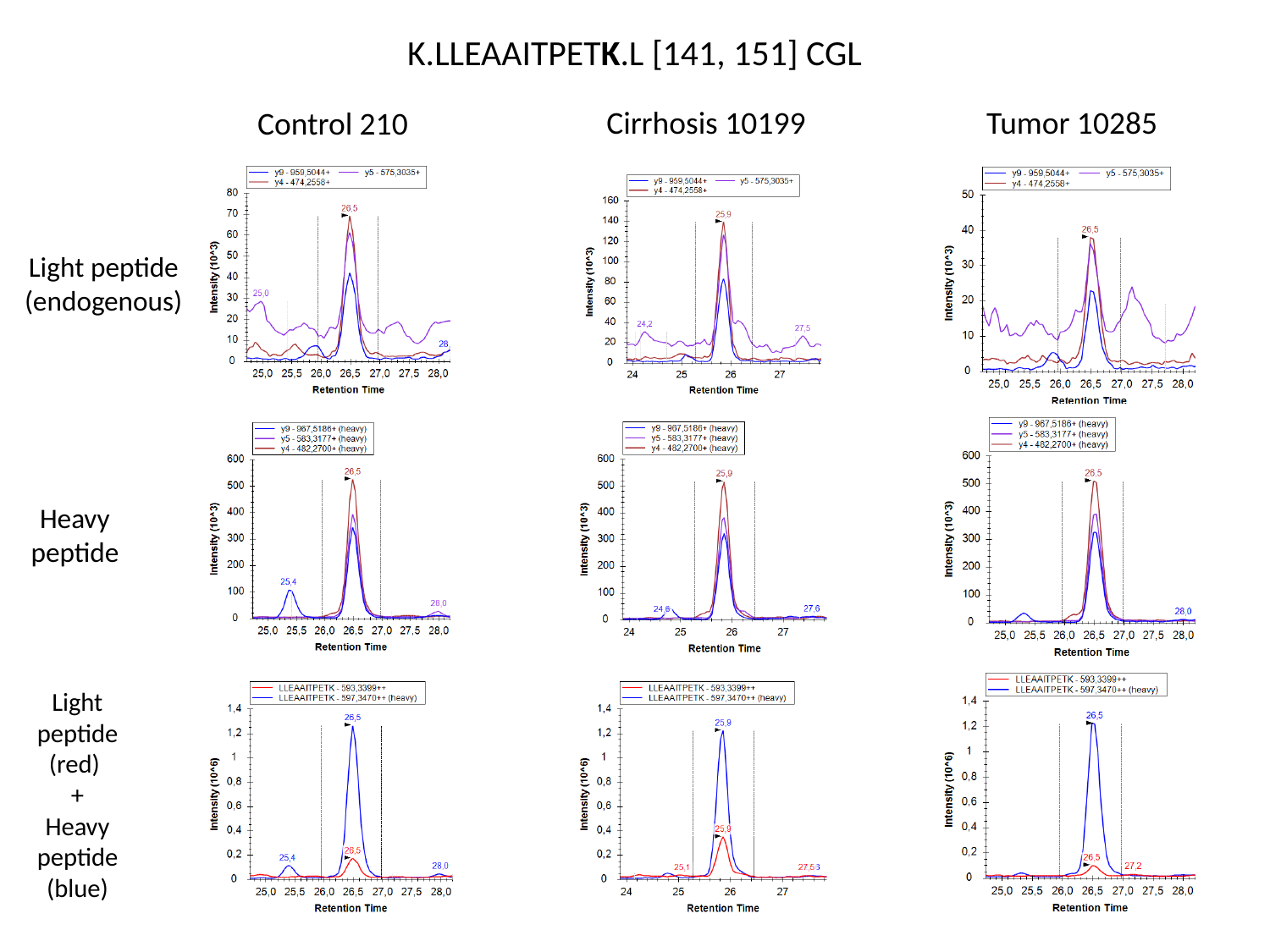

K.LLEAAITPETK.L [141, 151] CGL
Control 210
Cirrhosis 10199
Tumor 10285
Light peptide
(endogenous)
Heavy peptide
Light peptide (red)
+
Heavy peptide (blue)

## Slide 10
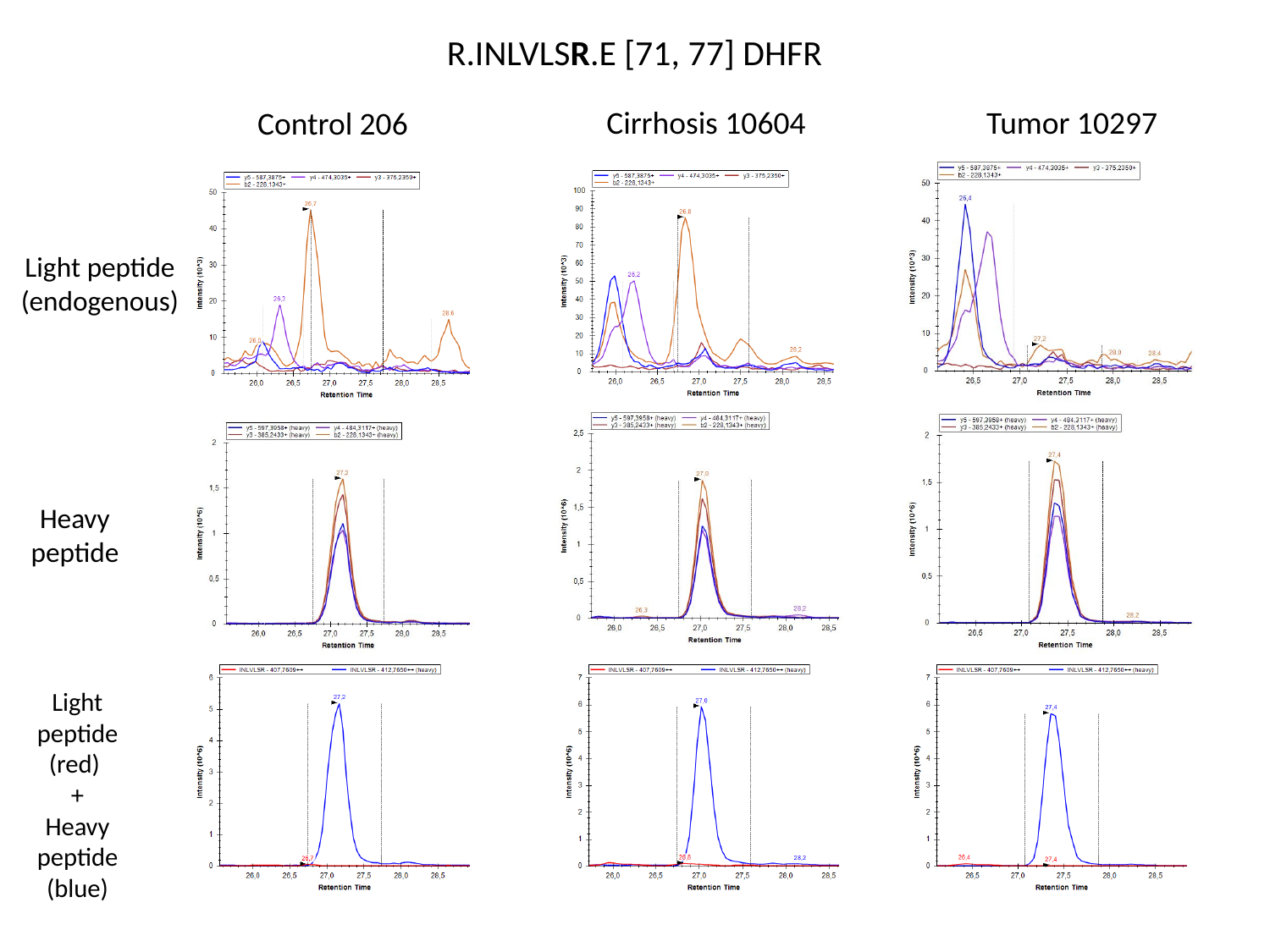

R.INLVLSR.E [71, 77] DHFR
Control 206
Cirrhosis 10604
Tumor 10297
Light peptide
(endogenous)
Heavy peptide
Light peptide (red)
+
Heavy peptide (blue)

## Slide 11
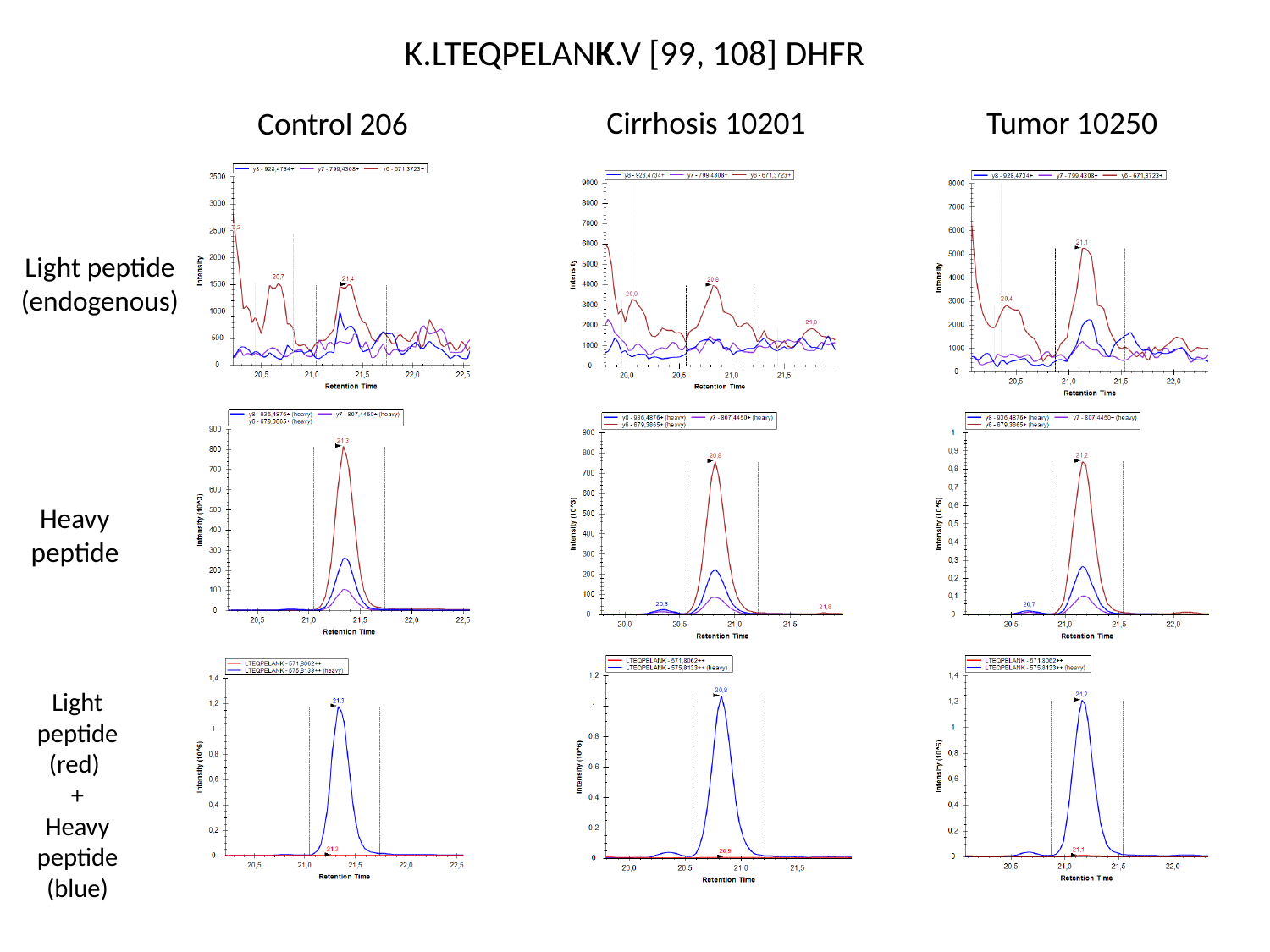

K.LTEQPELANK.V [99, 108] DHFR
Control 206
Cirrhosis 10201
Tumor 10250
Light peptide
(endogenous)
Heavy peptide
Light peptide (red)
+
Heavy peptide (blue)

## Slide 12
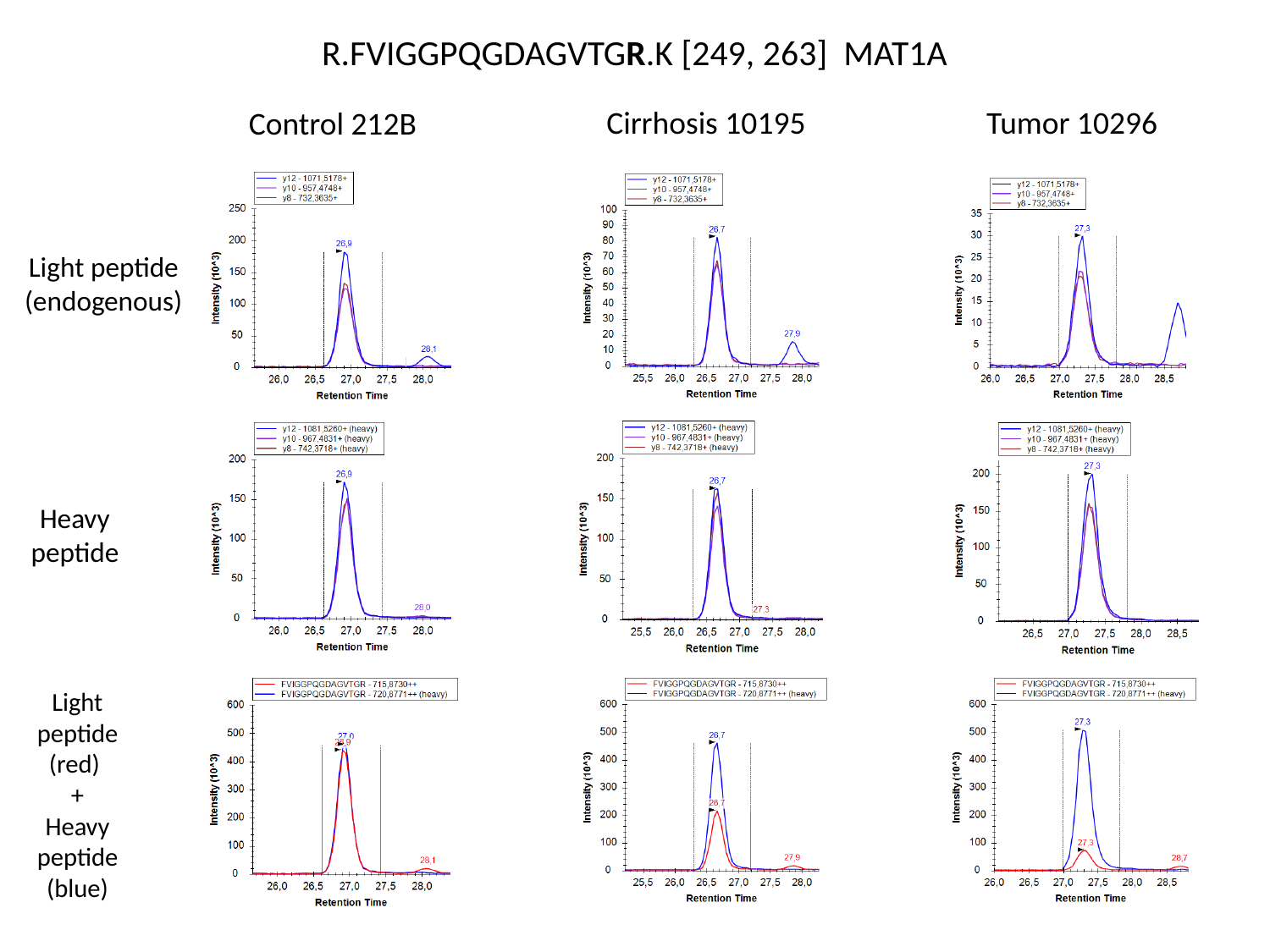

R.FVIGGPQGDAGVTGR.K [249, 263] MAT1A
Control 212B
Cirrhosis 10195
Tumor 10296
Light peptide
(endogenous)
Heavy peptide
Light peptide (red)
+
Heavy peptide (blue)

## Slide 13
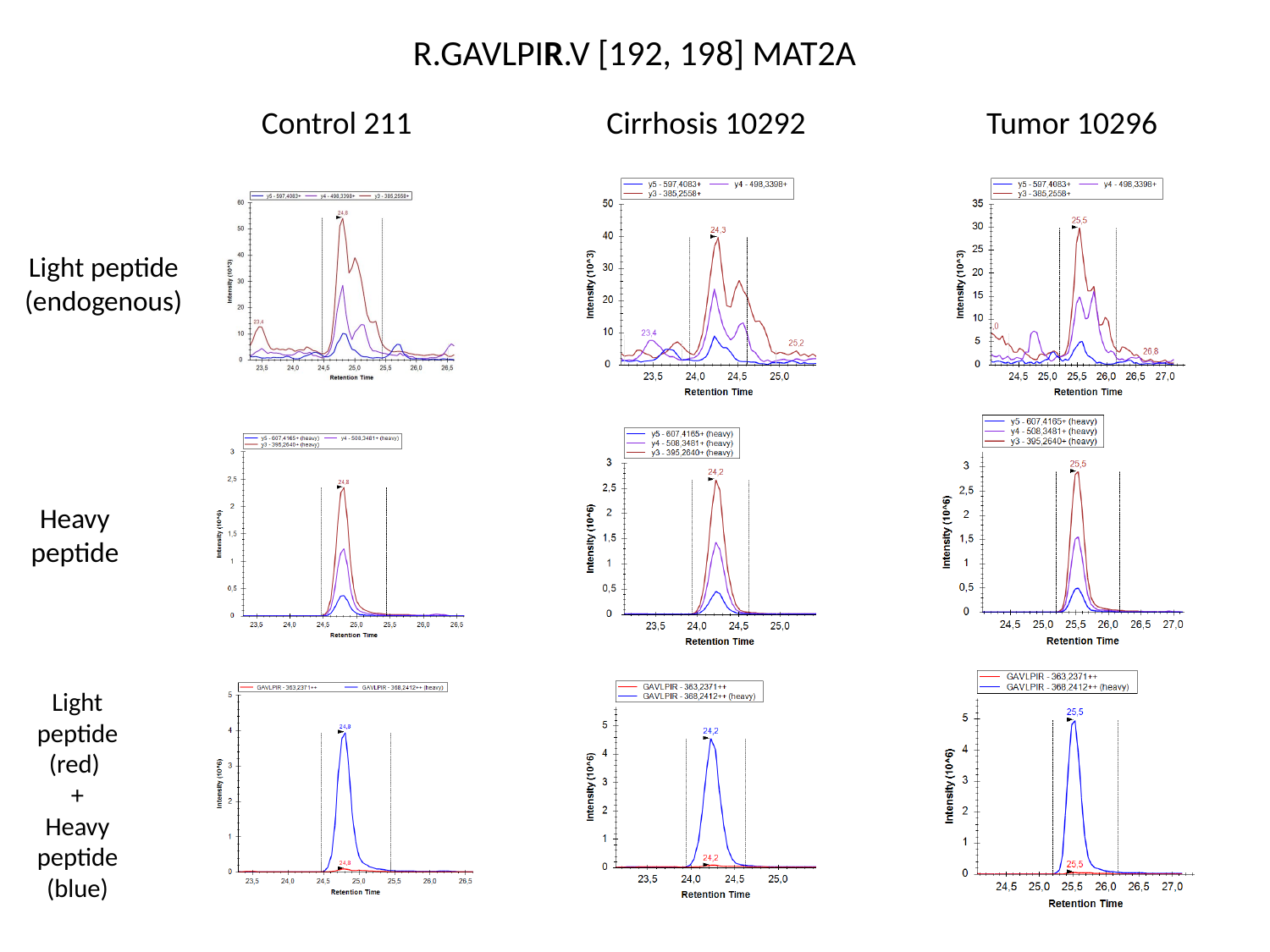

R.GAVLPIR.V [192, 198] MAT2A
Control 211
Cirrhosis 10292
Tumor 10296
Light peptide
(endogenous)
Heavy peptide
Light peptide (red)
+
Heavy peptide (blue)

## Slide 14
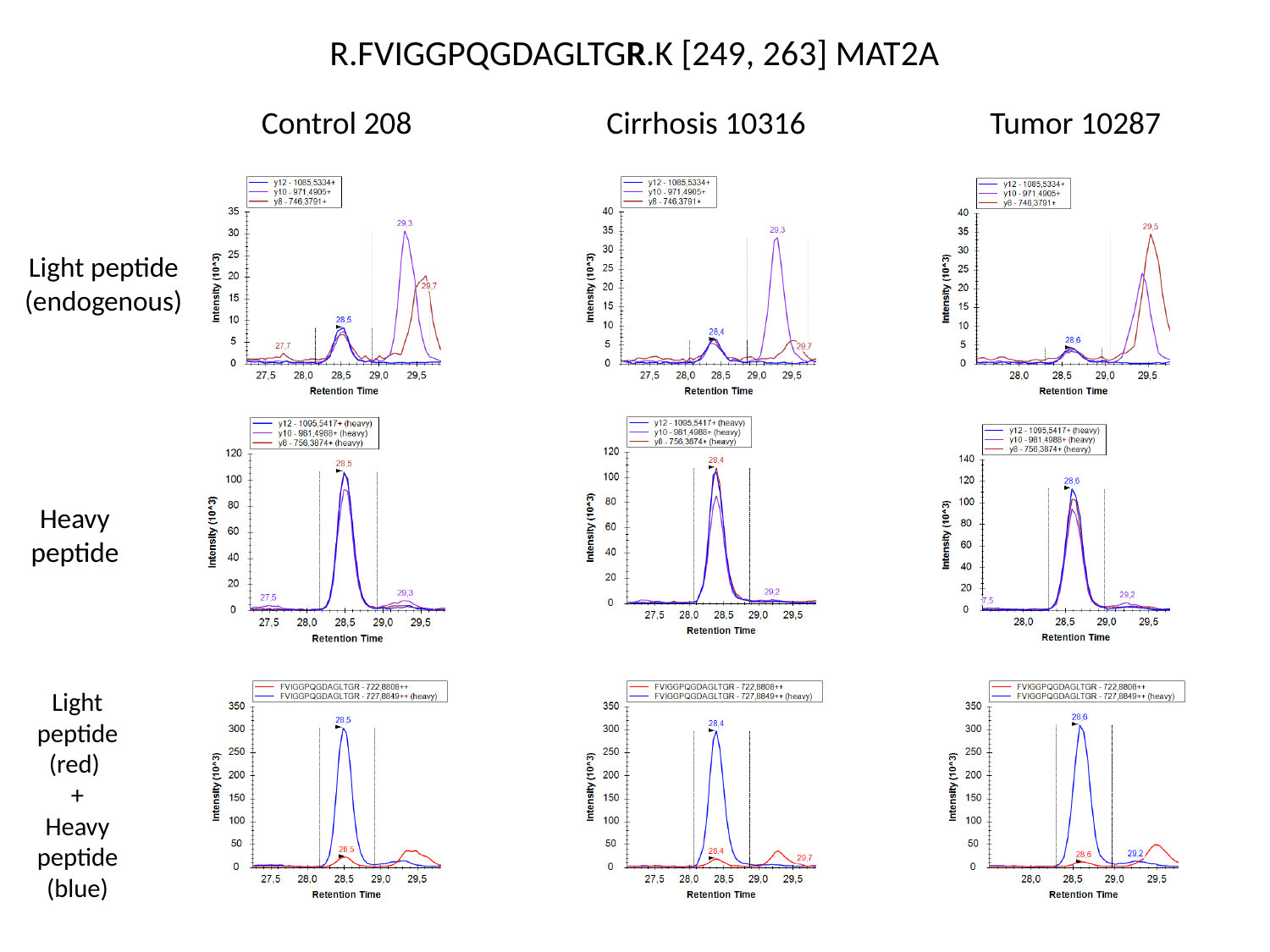

R.FVIGGPQGDAGLTGR.K [249, 263] MAT2A
Control 208
Cirrhosis 10316
Tumor 10287
Light peptide
(endogenous)
Heavy peptide
Light peptide (red)
+
Heavy peptide (blue)

## Slide 15
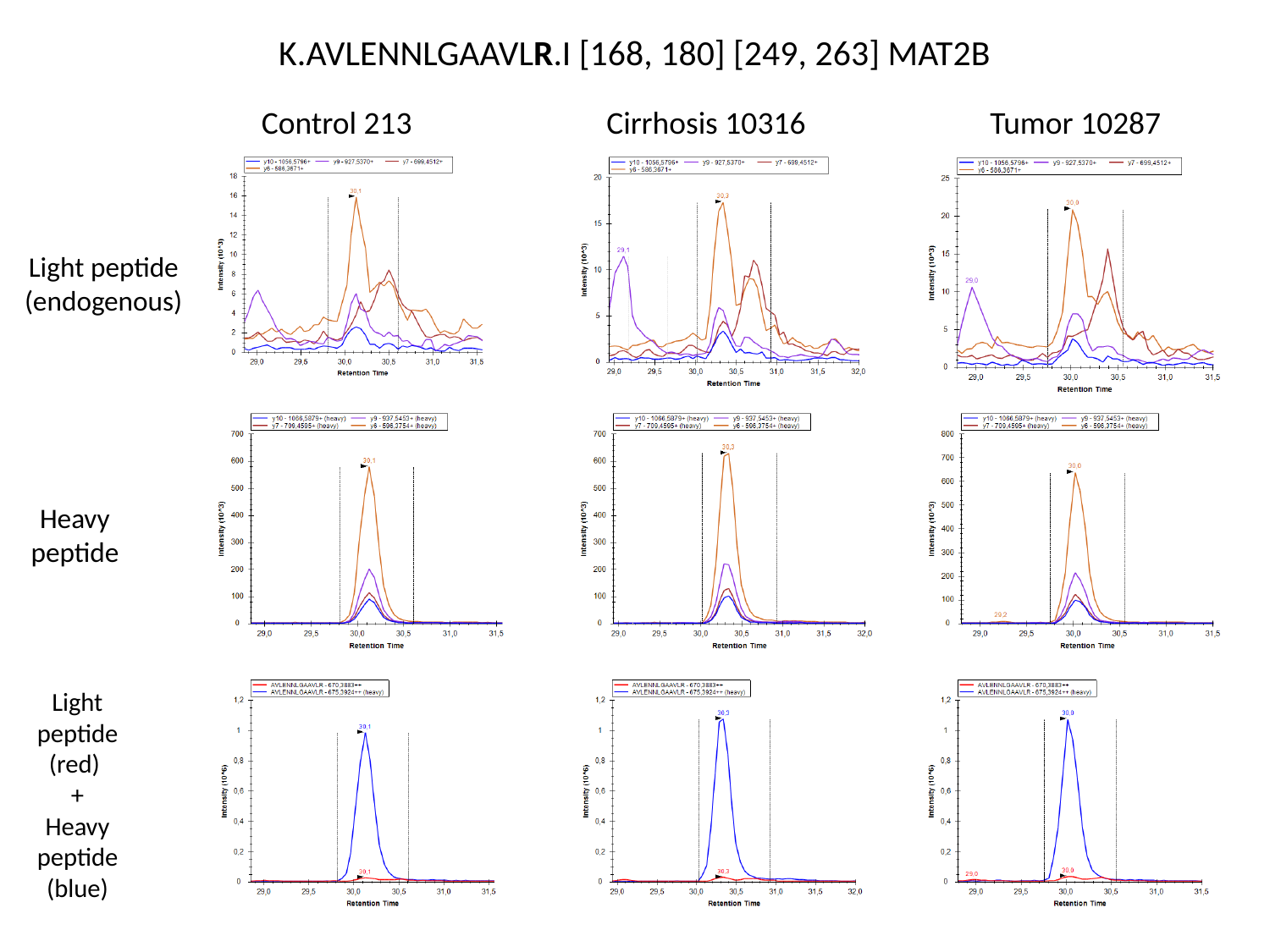

K.AVLENNLGAAVLR.I [168, 180] [249, 263] MAT2B
Control 213
Cirrhosis 10316
Tumor 10287
Light peptide
(endogenous)
Heavy peptide
Light peptide (red)
+
Heavy peptide (blue)

## Slide 16
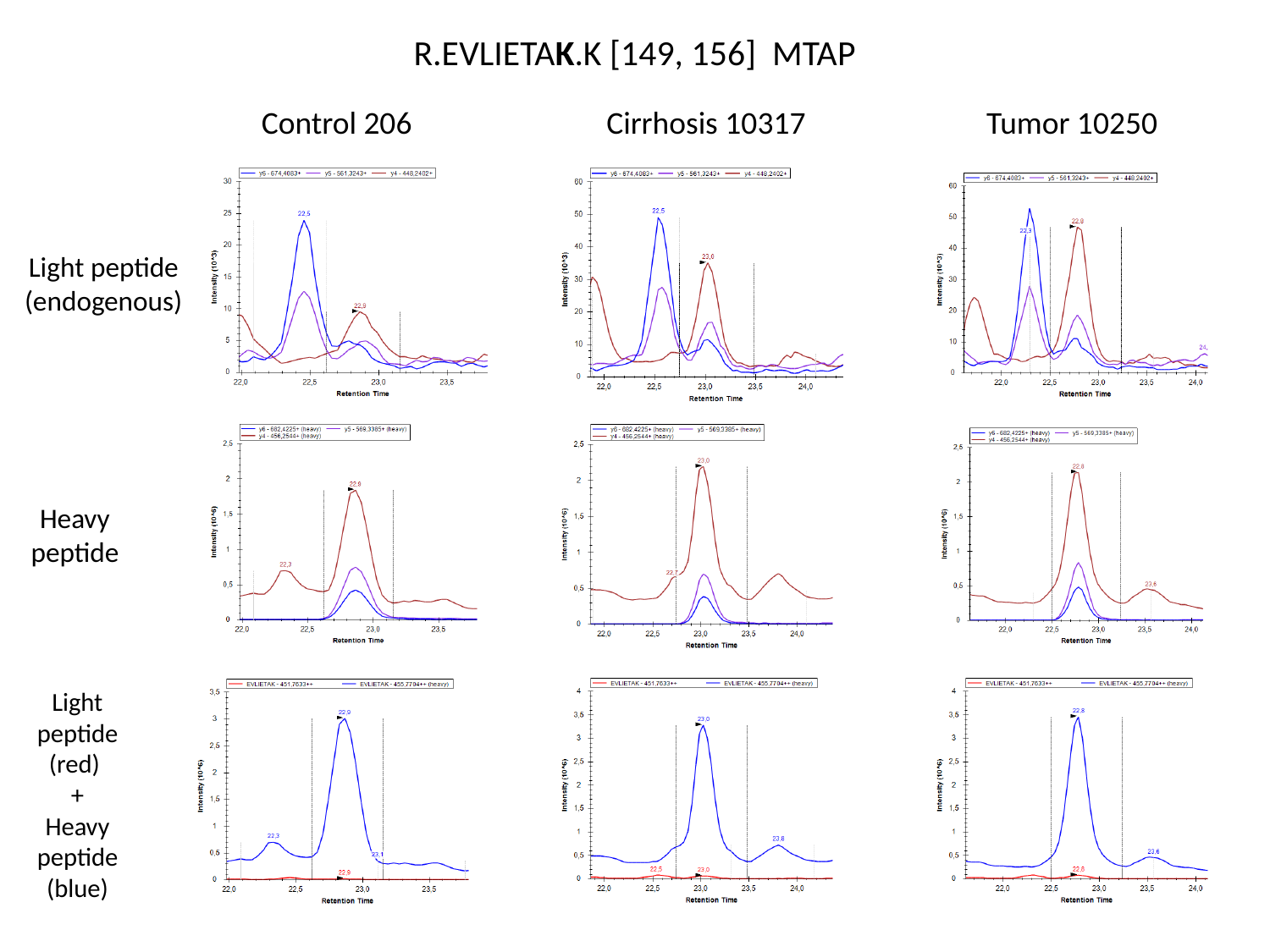

R.EVLIETAK.K [149, 156] MTAP
Control 206
Cirrhosis 10317
Tumor 10250
Light peptide
(endogenous)
Heavy peptide
Light peptide (red)
+
Heavy peptide (blue)

## Slide 17
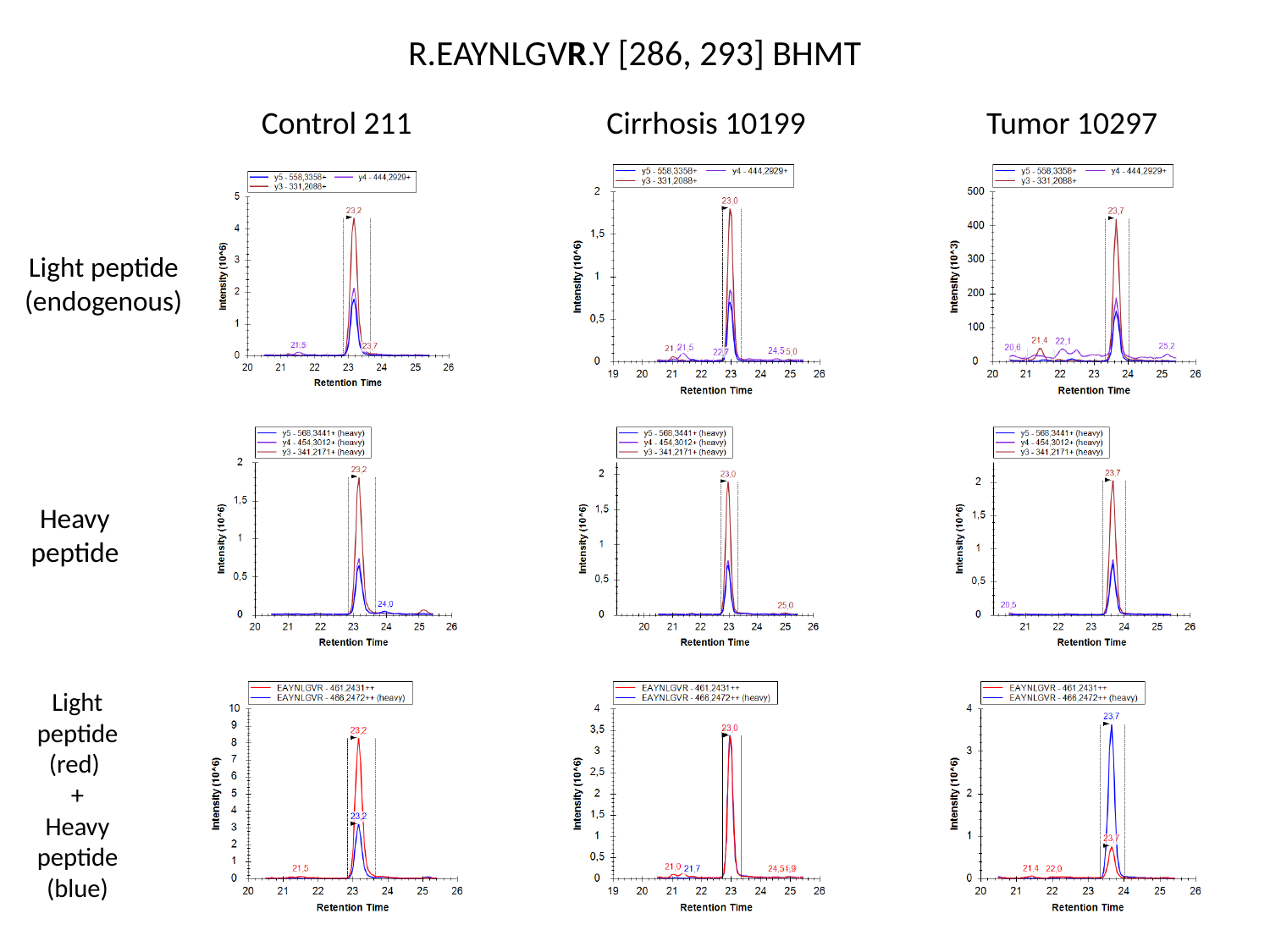

R.EAYNLGVR.Y [286, 293] BHMT
Control 211
Cirrhosis 10199
Tumor 10297
Light peptide
(endogenous)
Heavy peptide
Light peptide (red)
+
Heavy peptide (blue)

## Slide 18
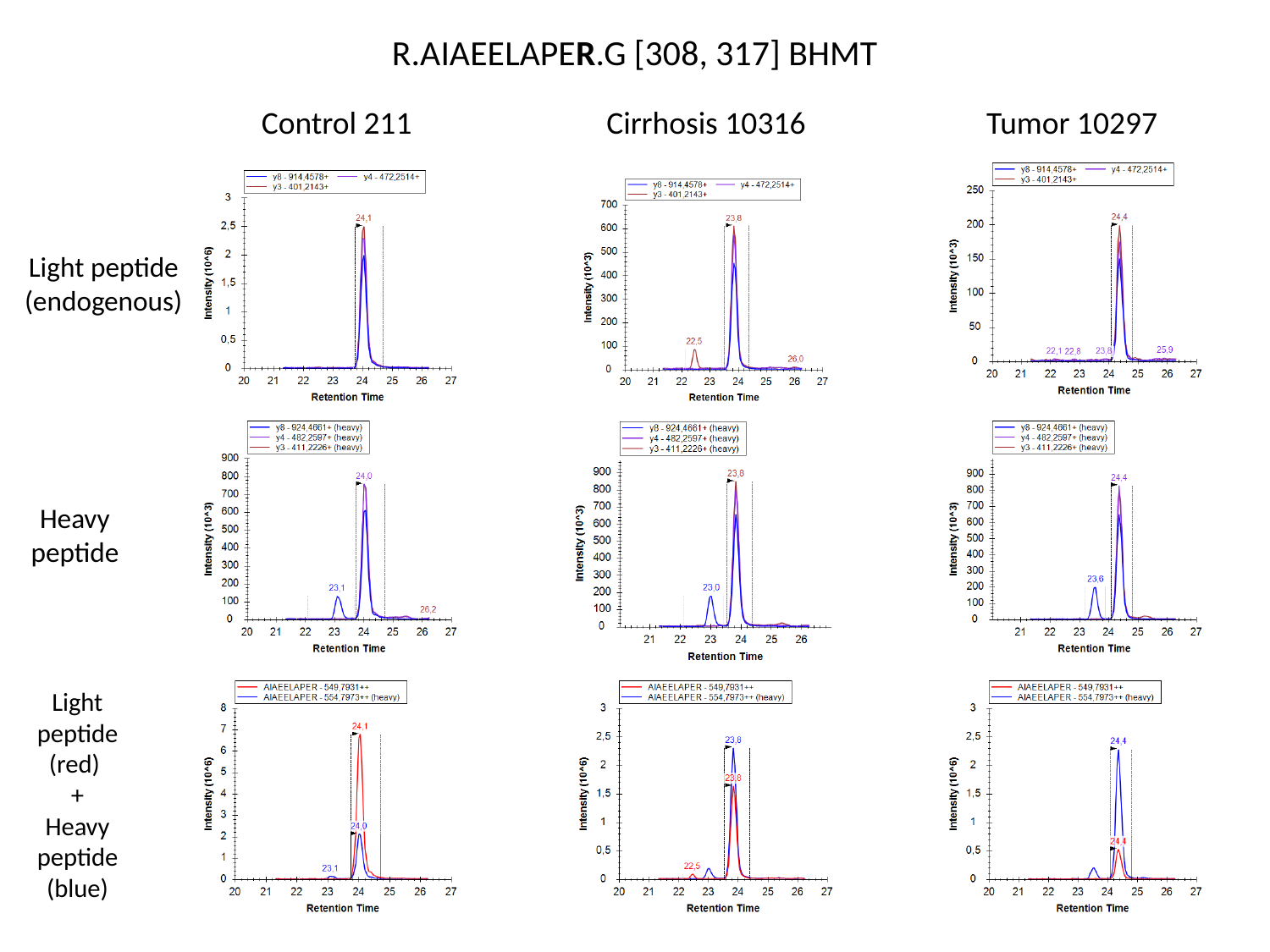

R.AIAEELAPER.G [308, 317] BHMT
Control 211
Cirrhosis 10316
Tumor 10297
Light peptide
(endogenous)
Heavy peptide
Light peptide (red)
+
Heavy peptide (blue)

## Slide 19
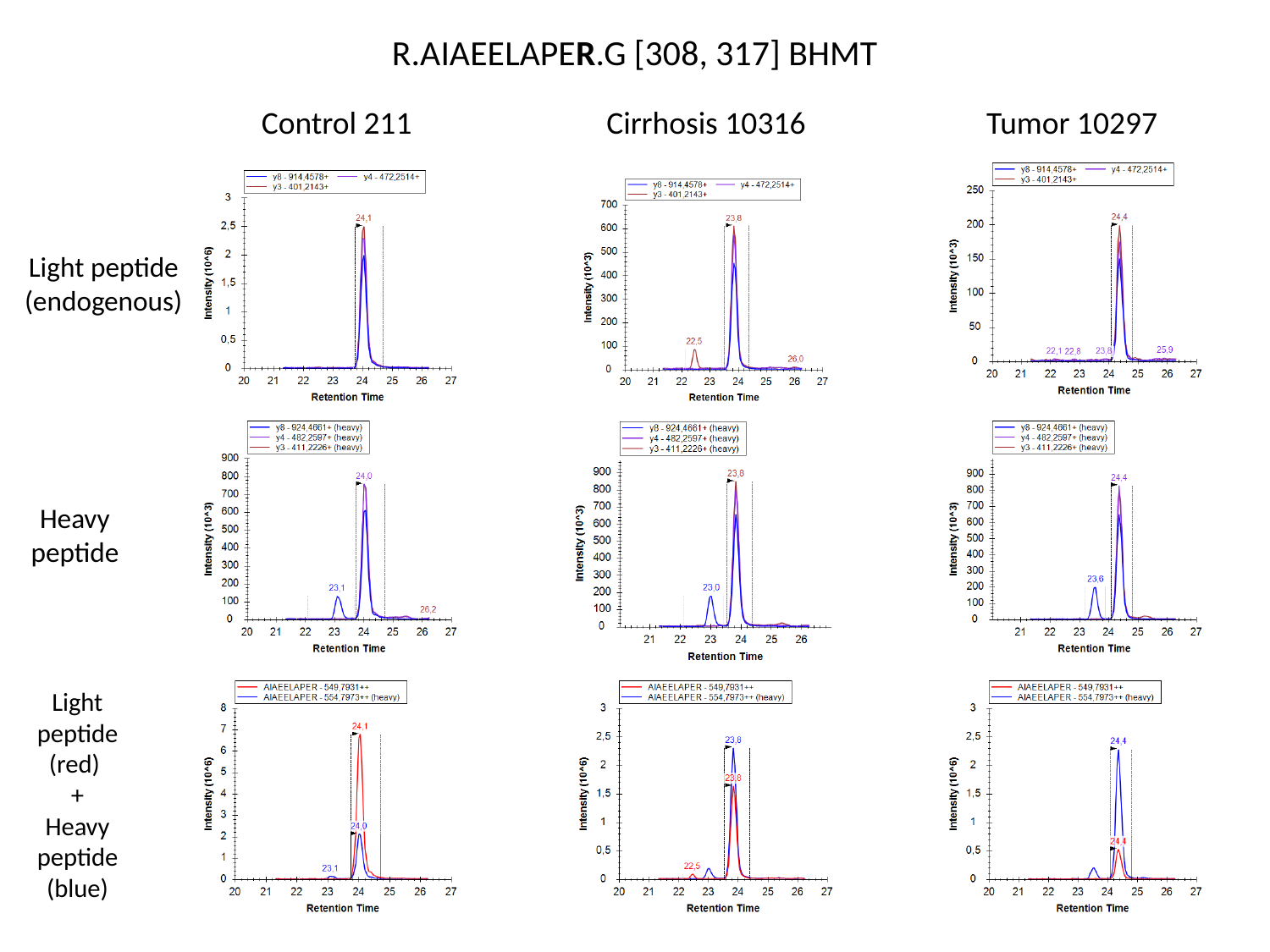

R.AIAEELAPER.G [308, 317] BHMT
Control 211
Cirrhosis 10316
Tumor 10297
Light peptide
(endogenous)
Heavy peptide
Light peptide (red)
+
Heavy peptide (blue)

## Slide 20
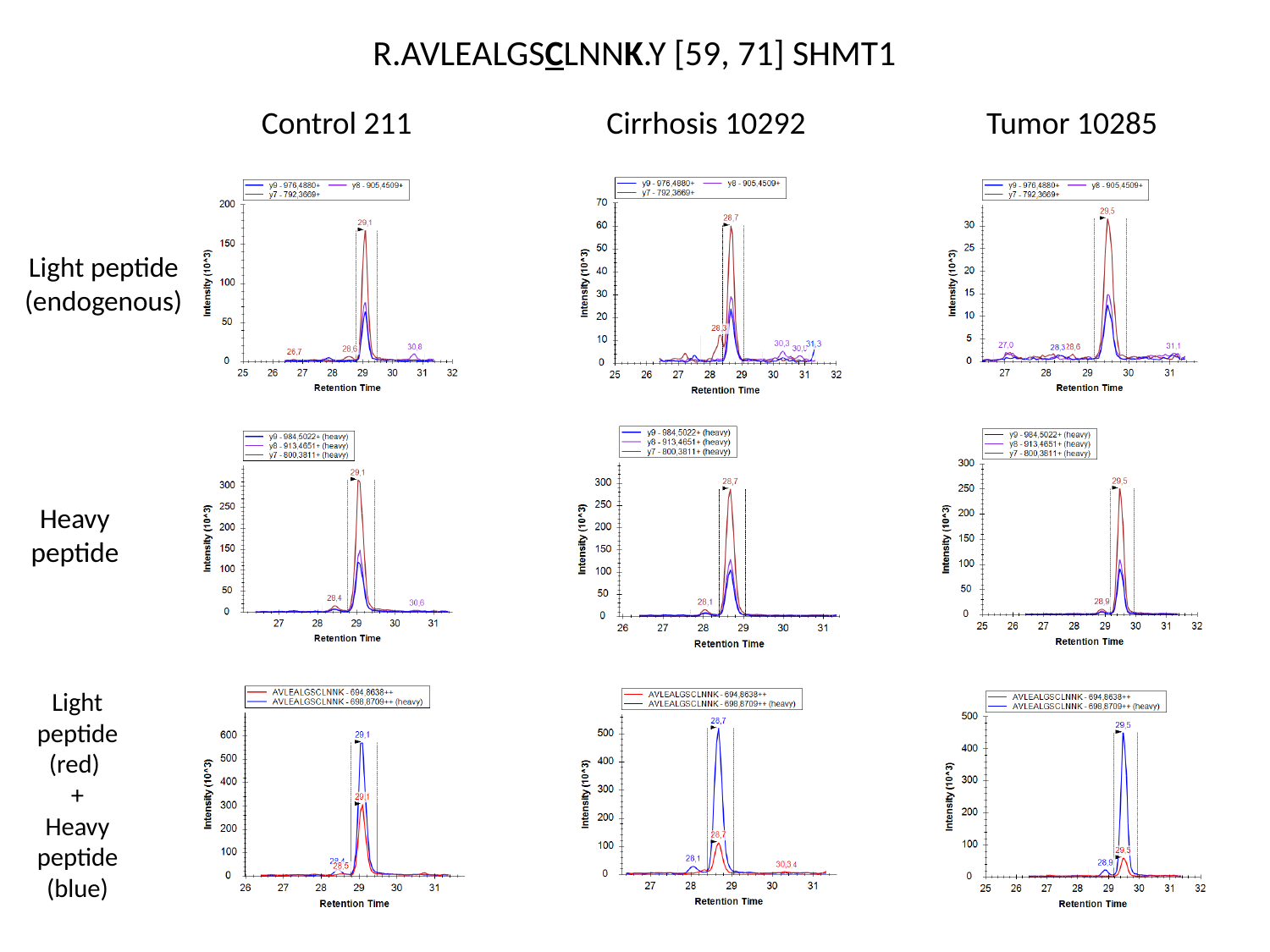

R.AVLEALGSCLNNK.Y [59, 71] SHMT1
Control 211
Cirrhosis 10292
Tumor 10285
Light peptide
(endogenous)
Heavy peptide
Light peptide (red)
+
Heavy peptide (blue)

## Slide 21
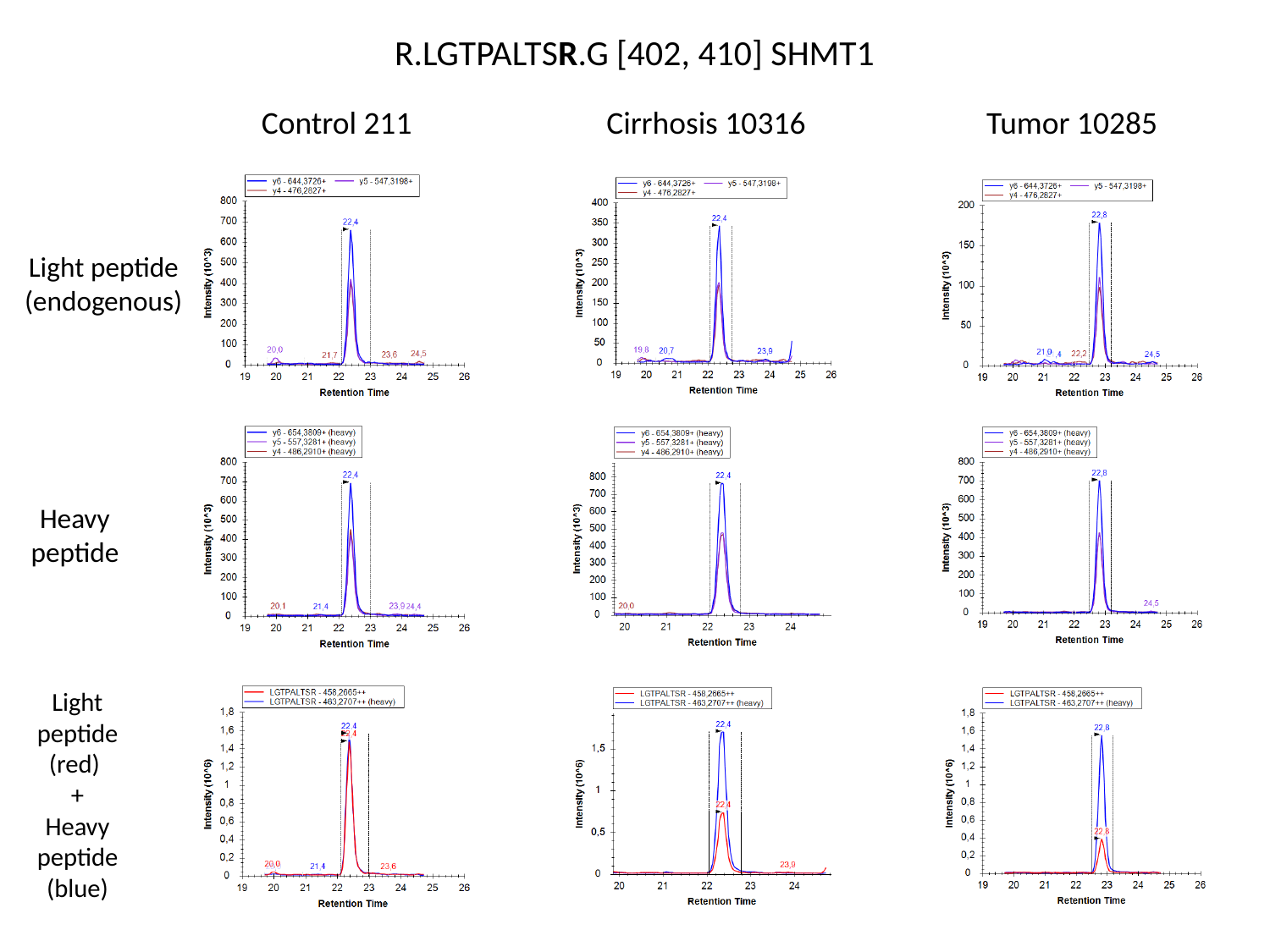

R.LGTPALTSR.G [402, 410] SHMT1
Control 211
Cirrhosis 10316
Tumor 10285
Light peptide
(endogenous)
Heavy peptide
Light peptide (red)
+
Heavy peptide (blue)

## Slide 22
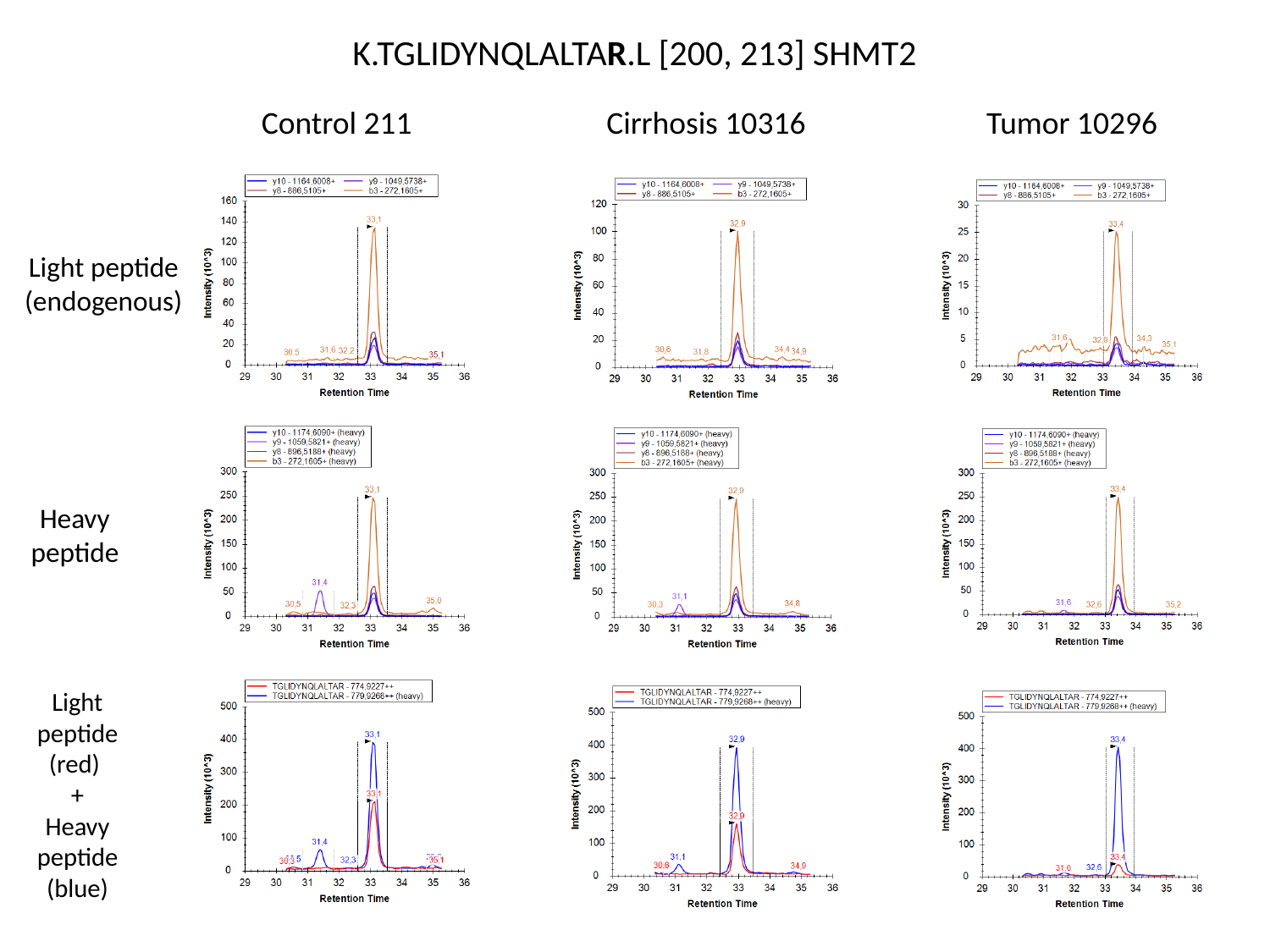

K.TGLIDYNQLALTAR.L [200, 213] SHMT2
Control 211
Cirrhosis 10316
Tumor 10296
Light peptide
(endogenous)
Heavy peptide
Light peptide (red)
+
Heavy peptide (blue)

## Slide 23
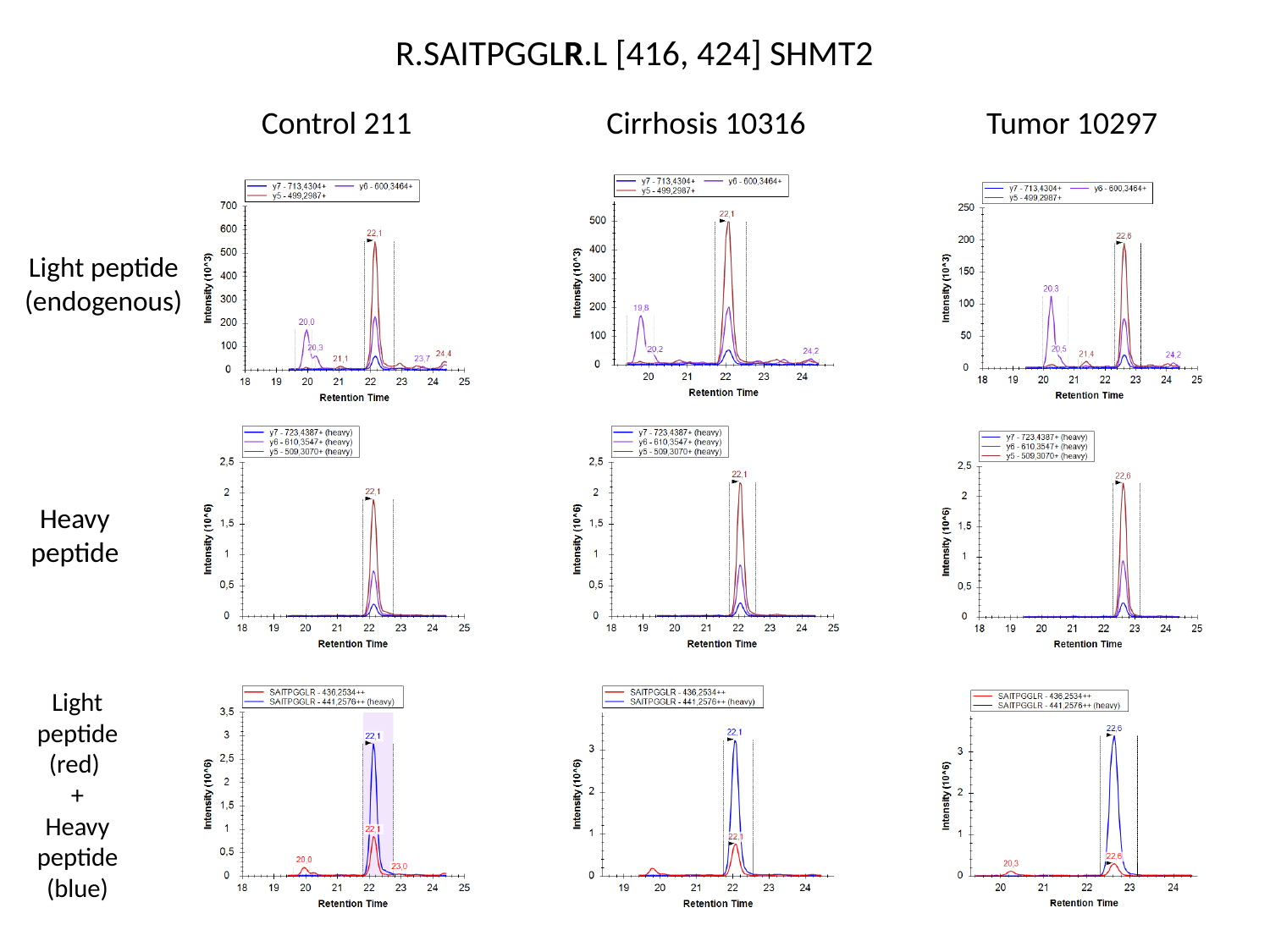

R.SAITPGGLR.L [416, 424] SHMT2
Control 211
Cirrhosis 10316
Tumor 10297
Light peptide
(endogenous)
Heavy peptide
Light peptide (red)
+
Heavy peptide (blue)

## Slide 24
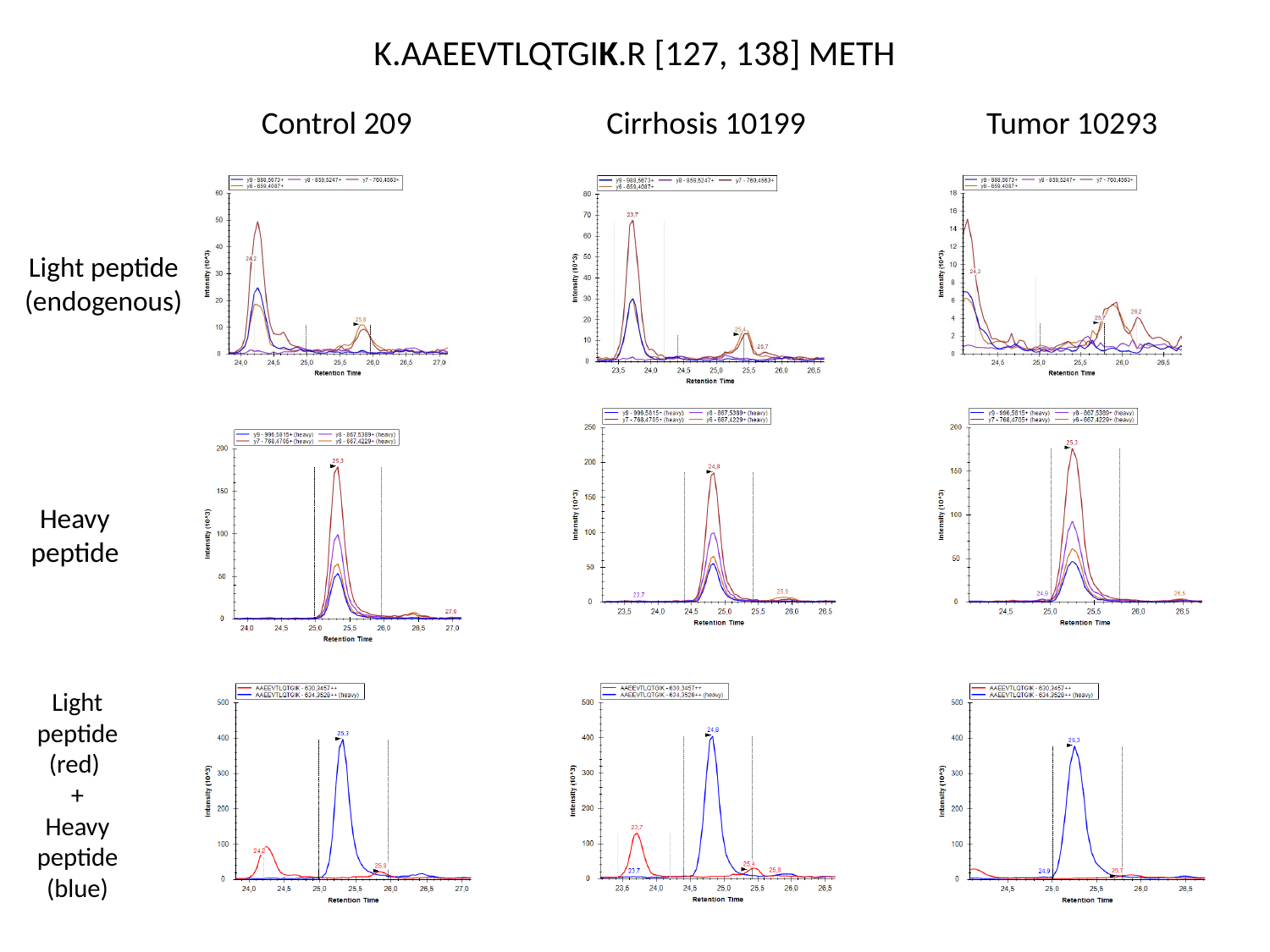

K.AAEEVTLQTGIK.R [127, 138] METH
Control 209
Cirrhosis 10199
Tumor 10293
Light peptide
(endogenous)
Heavy peptide
Light peptide (red)
+
Heavy peptide (blue)

## Slide 25
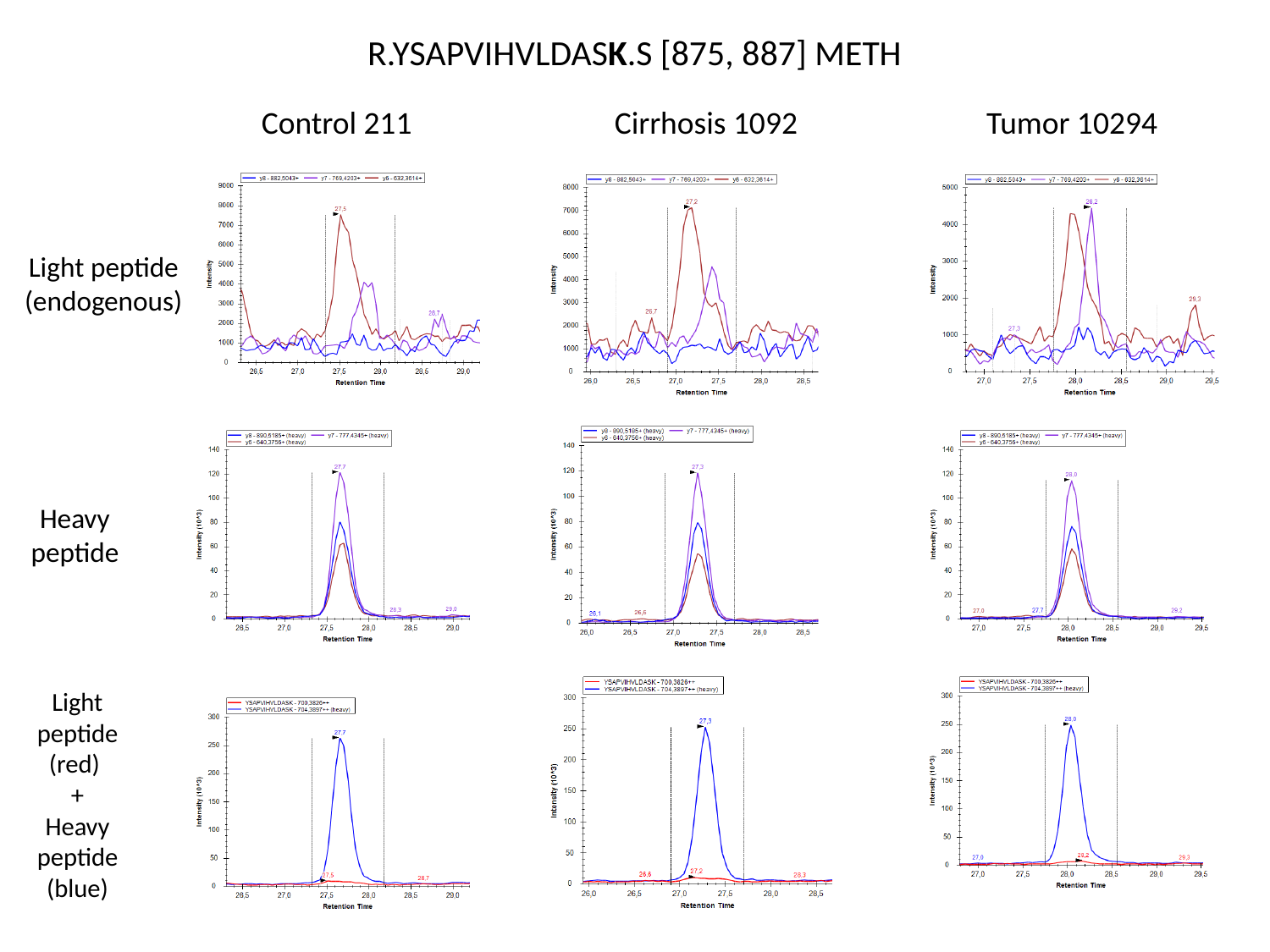

R.YSAPVIHVLDASK.S [875, 887] METH
Control 211
Cirrhosis 1092
Tumor 10294
Light peptide
(endogenous)
Heavy peptide
Light peptide (red)
+
Heavy peptide (blue)

## Slide 26
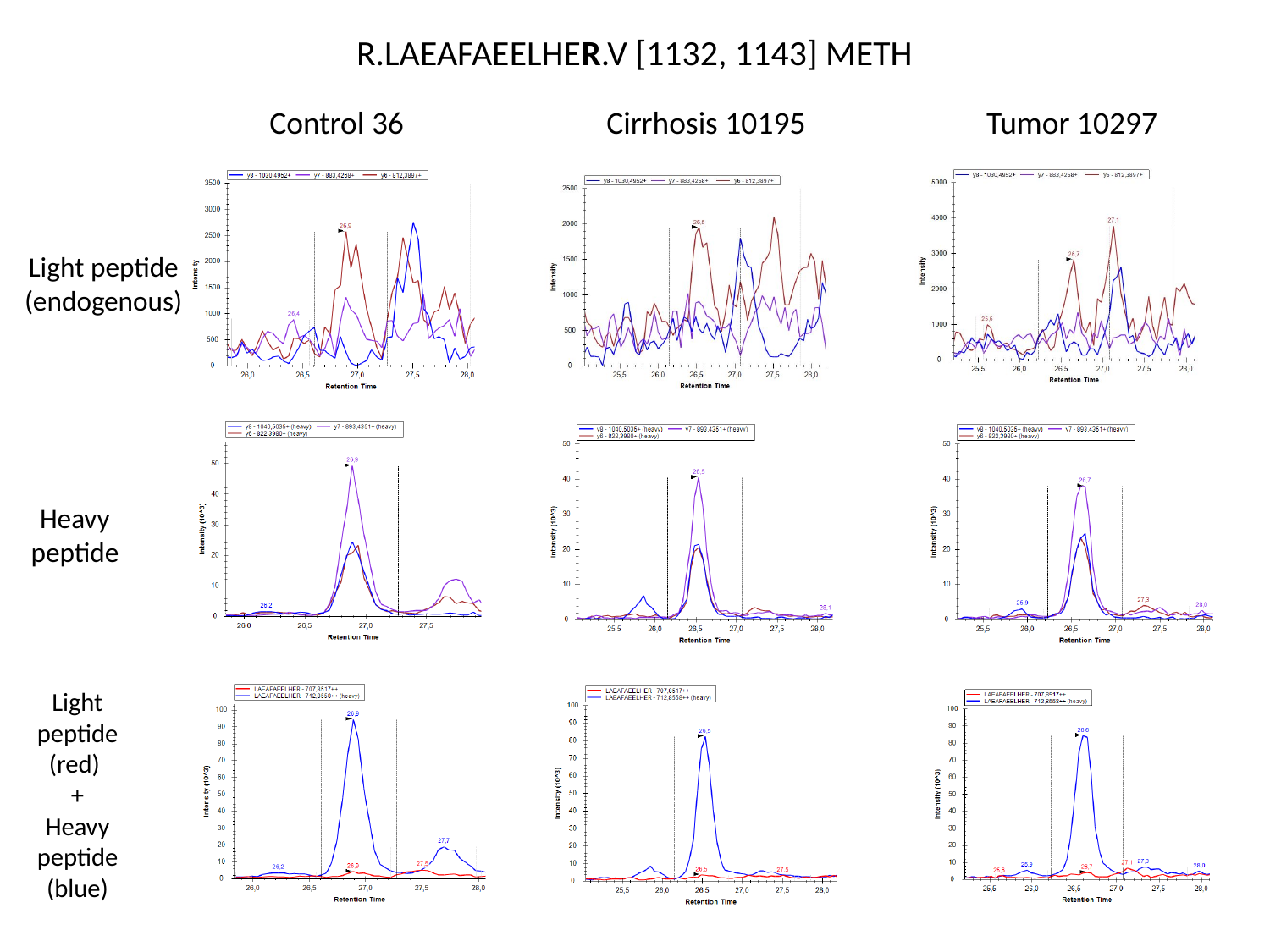

R.LAEAFAEELHER.V [1132, 1143] METH
Control 36
Cirrhosis 10195
Tumor 10297
Light peptide
(endogenous)
Heavy peptide
Light peptide (red)
+
Heavy peptide (blue)
